# Functional consequences of genetic variations in DgoR, a GntR/FadR family transcriptional repressor of D-galactonate metabolism in *Escherichia coli*

**DOI:** 10.1128/jb.00103-25

**Published:** 2025-06-11

**Authors:** Swati Singh, Rajesh Mishra, Richa Ashok Kakkar, Shivam Singla, Akhil Pratap, Gaurav Sharma, Monika Sharma, Rachna Chaba

**Affiliations:** 1 Department of Biological Sciences, Indian Institute of Science Education and Research 124268, Mohali, Punjab, India; 2 Department of Biotechnology, Indian Institute of Technology Hyderabad, Sangareddy, Telangana, India; 3 Biological Systems Engineering, Plaksha University, Mohali, Punjab, India; University of Virginia School of Medicine, Charlottesville, Virginia, USA

**Keywords:** carbohydrate metabolism, effector, gene regulation, ligand, sugar acid, transcription factors

## Abstract

Genetic variations in transcriptional regulators (TRs) of metabolic loci can influence host-bacterial interactions by affecting carbon utilization. Although the metabolism of sugar acids, including D-galactonate, is extensively implicated in the colonization and virulence of enteric bacteria, there has been no investigation on the extent of variations in their pathway-specific TRs. DgoR, the TR of D-galactonate metabolism, is the best-characterized GntR/FadR family sugar acid TR in enteric bacteria, recognized by the presence of an N-terminal winged helix-turn-helix DNA-binding domain and a C-terminal effector-binding and oligomerization (E-O) domain connected by a linker. Here, we examined 340 *Escherichia coli* isolates for variations in *dgoR* and studied their effect on repressor function. Genetic and biochemical studies identified variants with a partial loss of DNA-binding ability (P24L and A152E) and a decreased response to D-galactonate (R71C and P92L). Because the linker residue R71C resulted in a reduced response to D-galactonate and the E-O domain residue A152E led to a DNA binding defect, we performed simulations to probe their altered allosteric behavior. We observed that the correlation patterns, dynamics, and networks of the variants are indeed distinct from the wild type. Importantly, corroborating their repressor function, R71C and A152E variations impacted the growth of natural isolates in D-galactonate. Alignment-based variation detection across all *E. coli* and Enterobacterales identical protein group data sets revealed less prevalence of these four variations. Collectively, the present study highlights the need for a thorough analysis of the effect of variations in sugar acid TRs on repressor function and their effect on host-bacterial interactions.

**IMPORTANCE:**

Sugar acids are used as carbon sources by enteric bacteria, both commensals and pathogens, with numerous studies highlighting their importance in host-bacterial interactions. Here, taking *Escherichia coli* DgoR, the transcriptional regulator (TR) of D-galactonate metabolism, as a representative, we showed that genetic variations in sugar acid TRs can affect their function and impact the utilization of these carbon sources by natural isolates. As the ability to use limiting nutrients enables bacteria to compete with the complex microbial community of the host, our study emphasizes the need for a comprehensive analysis of variations in sugar acid TRs to determine whether they influence the competition. These studies can help envision approaches for promoting the growth of commensals to eliminate their pathogenic counterparts.

## INTRODUCTION

Sugar acids, the oxidized derivatives of sugars, are extensively used as a nutrient source by enteric bacteria. These carbon sources have wide natural prevalence: they are constituents of the plant cell wall and animal tissues (1); they are intermediates of sugar metabolism in several bacteria, fungi, and higher organisms, including humans (2–7); microbiota liberates sugar acids from polysaccharides ingested by the host and the mucosal layer of the gut epithelium (8–10); and antibiotic treatment induces host-mediated oxidation of sugars in the gut (11). Enteric bacteria degrade sugar acids via the Entner-Doudoroff or Ashwell pathway (12), which is mainly regulated by GntR/FadR family transcriptional regulators (TRs), characterized by the presence of an N-terminal winged helix-turn-helix (wHTH) DNA-binding domain (Pfam, PF00392) and all α-helical C-terminal effector-binding and oligomerization (E-O) domain, connected by a linker (13–17). These TRs usually repress metabolic genes, and the repression is relieved upon binding cognate effectors, which are substrates, pathway intermediates, or both (18–25). Only a few GntR/FadR family sugar acid TRs from enteric bacteria have been characterized, namely, DgoR (repressor of D-galactonate metabolism), ExuR (repressor of D-galacturonate and D-glucuronate metabolism), and UxuR (repressor of D-fructuronate and D-glucuronate metabolism) from *Escherichia coli*. DNA-binding characteristics and effectors are known for all three TRs; however, the effector-binding cavity has been identified only for DgoR (18, 22–24, 26, 27). Furthermore, among the three sugar acid TRs, only the crystal structure of the C-terminal E-O domain of DgoR is known (27), and the allosteric mechanism that governs the effector-mediated DNA release has also been reported only for DgoR (28).

Sugar acids are highly implicated in the interaction of enteric bacteria with their hosts. For example, *E. coli* fecal isolates use D-gluconate for intestinal colonization (10); D-galactarate and D-glucarate metabolism enables expansion of *Salmonella enterica* serovar Typhimurium in the gut (11); *Citrobacter rodentium* uses D-glucuronate for intestinal colonization (29), and both *C. rodentium* and enterohemorrhagic *E. coli* (EHEC) use D-galacturonate for intestinal colonization and as a virulence signal (30). Notably, numerous studies in the last couple of decades have suggested that D-galactonate metabolism plays an important role in the physiology of enteric bacteria inhabiting diverse habitats. For instance, the *dgo* operon (D-galactonate operon) involved in D-galactonate metabolism is upregulated in *Salmonella enterica* strains grown in egg white, epithelial cells, the gastrointestinal tract, macrophages, and soft-rotted leaves and in an asymptomatic *E. coli* strain cultured in human urine (31–36). Furthermore, DgoT, the transporter of D-galactonate, is a virulence determinant of *S. enterica* serovar Choleraesuis in pigs (37). Finally, in multiple long-term evolution experiments where *E. coli* isolates were introduced in the mouse gut, inactivating mutations were mainly recovered in *dgoR*, indicating that D-galactonate utilization enables *E. coli* adaptation in the mammalian gut (38–40).

As part of the complex microbial community, bacteria must be capable of using limiting nutrients for successful colonization within the host and causing infections. Metabolic diversity impacts the fitness, virulence, host range, and tropism of even the closely related strains, which often results from variations in metabolic loci and regulatory elements (41–44). Importantly, several instances of single nucleotide polymorphisms in TRs of carbon metabolism are known to affect the utilization of nutrient sources, which dramatically impacts the outcome of host-bacterial interactions (41–43). Although sugar acid metabolism has been widely implicated in the colonization and virulence of enteric bacteria, there has been no investigation on the extent of genetic variations in their pathway-specific TRs and their effect on carbon source utilization. Considering the physiological importance of D-galactonate metabolism and that among all three characterized GntR/FadR sugar acid TRs in enteric bacteria, DgoR is the best-studied TR, here, we chose to examine the effect of genetic variations in *dgoR* on the growth of *E. coli* isolates in D-galactonate and gain mechanistic insights into how these variations affect the repressor function.

Previous work from our lab established DgoR as a GntR/FadR family TR and showed that DgoR forms dimers and represses the *dgo* operon by binding to two closely spaced inverted repeats (IR1 and IR2), which overlap the *dgo* promoter (Fig. S1). We also identified D-galactonate as the specific effector of DgoR and its binding cavity in the C-terminal E-O domain of the repressor (22, 26, 45). Furthermore, in the absence of the full-length APO, DNA-bound, and effector-bound structures, we performed molecular dynamics (MD) simulations of the modeled DgoR structure in different allosteric states and proposed the allosteric mechanism that governs the D-galactonate-mediated DNA release from DgoR (28).

**Fig 1:**
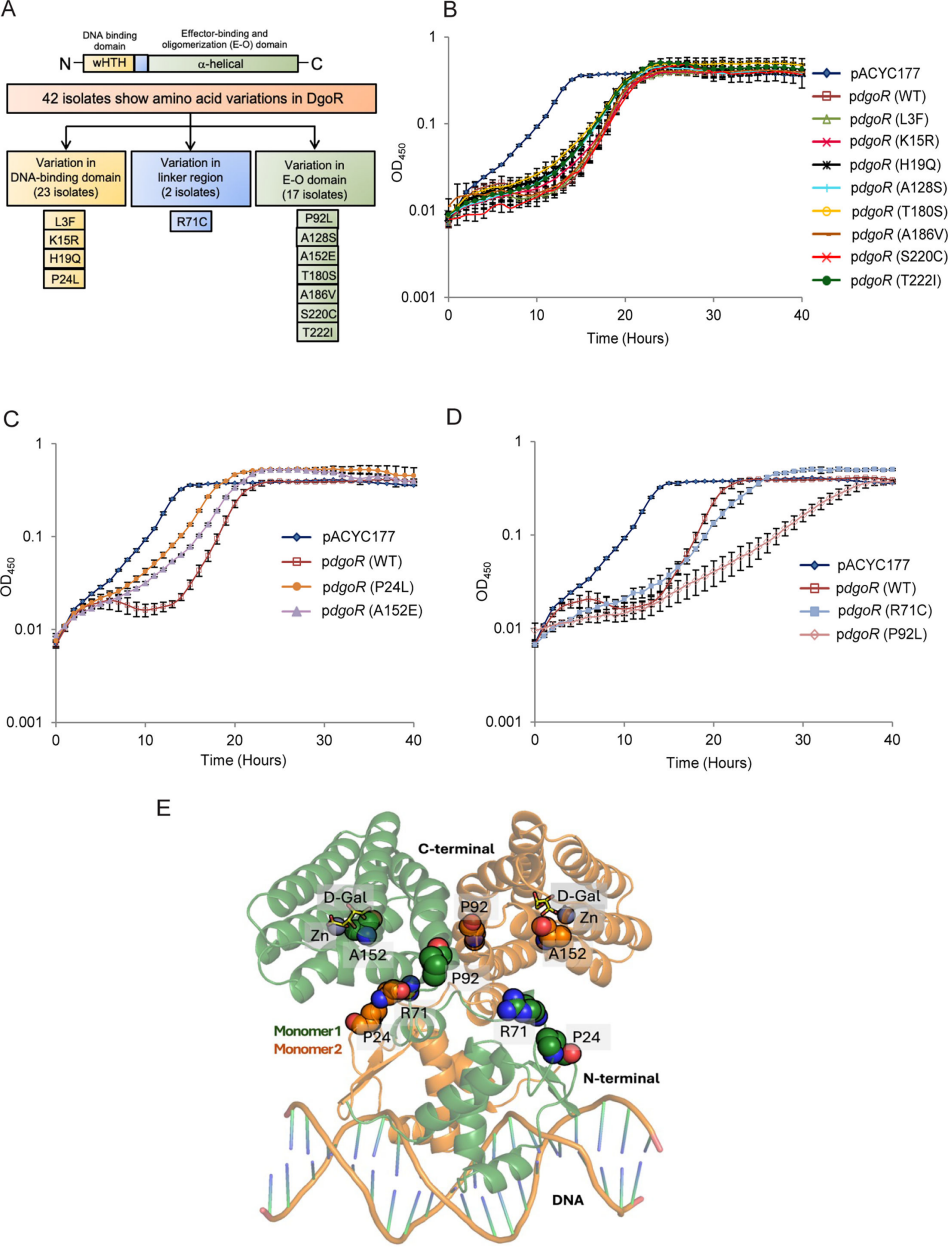
Of the 12 unique amino acid variations in DgoR among the natural isolates, four confer either fast or slow growth phenotype to BW in medium containing D-galactonate. (**A**) Schematic showing amino acid variations in DgoR among the natural *E. coli* isolates. A genetic reference panel of 340 sequenced natural *E. coli* isolates (46) was analyzed for variations in DgoR in comparison to the amino acid sequence of BW DgoR. Forty-two strains show 12 unique amino acid changes: four in the N-terminal wHTH DNA-binding domain, one in the linker region, and seven in the C-terminal E-O domain. (**B–D**) P24L and A152E variants confer fast growth phenotype, while R71C and P92L variants confer slow growth phenotype to BW in a medium containing D-galactonate. The plasmid pACYC177 and BW WT *dgoR* or its variants cloned in pACYC177 [p*dgoR*(WT), pBS13; p*dgoR*(L3F), pSW12; p*dgoR*(K15R), pSW13; p*dgoR*(H19Q), pSW16; p*dgoR*(P24L), pSW10; p*dgoR*(R71C), pSW28; p*dgoR*(P92L), pSW29; p*dgoR*(A128S), pSW11; p*dgoR*(A152E), pSW26; p*dgoR*(T180S), pSW8; p*dgoR*(A186V), pMP18; p*dgoR*(S220C), pMP19; and p*dgoR*(T222I), pSW15] were individually transformed in a BW *dgoR::kan* strain. The transformants were grown in a minimal medium supplemented with D-galactonate, and the OD_450_ was measured. The experiment was done four times, each with three technical replicates. A representative data set, with average (±SD) from technical replicates, is shown. (**B**) Variants that do not affect the growth of BW. (**C**) Variants that confer a fast growth phenotype to BW. (**D**) Variants that confer a slow growth phenotype to BW. Data shown for pACYC177 and p*dgoR*(WT) in panels B–D are the same. (**E**) DgoR dimeric model bound to DNA and D-galactonate, showing the location of P24, R71, P92, and A152 residues. A ribbon representation of the two monomers of DgoR is shown in orange and green colors. Modeled DNA is represented in orange elemental color. Amino acid residues are shown in spheres. D-galactonate (D-Gal) bound in the pocket is shown in yellow sticks, and the Zn ion is shown in a purple sphere.

Here, we searched a genetic reference panel of 340 sequenced natural *E. coli* isolates (46) and identified 42 isolates with 12 unique amino acid variations in DgoR (in comparison to DgoR of *E. coli* K-12 BW25113 [henceforth designated as BW]) (Fig. 1A; Table S1). Through genetic tests, we found that four variations either lead to a partial loss of DNA-binding ability (P24L and A152E) or decreased sensitivity to D-galactonate (R71C and P92L). Importantly, corroborating their effect on the repressor function of BW DgoR, the R71C and A152E variations led to slower and faster growth of natural isolates in D-galactonate, respectively. Biochemical assays showed that, consistent with its compromised inducibility, R71C has a decreased affinity for D-galactonate, and aligning with its reduced repressor ability, A152E has a decreased affinity for the *dgo* promoter. Given that R71C, located in the linker region, affects effector binding, and A152E, present in the E-O domain, affects DNA binding, using MD simulations, we probed their impact on allosteric communication in DgoR. The distinct correlation patterns, dynamics, and networks observed in variants in response to DNA and effector indicated the differences in their structural and functional behavior compared to the wild type (WT). Collectively, the correlation between *in vivo* phenotypes, *in vitro* studies, and MD simulations strengthens the narrative of how specific amino acid variations modulate the function of DgoR.

## RESULTS

### DgoR variants are partially compromised for repression (P24L and A152E) or for responding to effector (R71C and P92L)

Among the 12 unique amino acid variations in DgoR that we identified in sequenced natural isolates (46), four are located in the DNA-binding domain, one is located in the linker, and seven are present in the E-O domain (Fig. 1A; Table S1). Using BW DgoR as a reference, we investigated whether these variations affect the functionality of the repressor. For this, we first compared the growth of BW ∆*dgoR* expressing the DgoR variants harboring these amino acid changes with cells expressing WT DgoR in a minimal medium containing D-galactonate as the sole carbon source. All proteins were expressed from the native *dgo* promoter of BW cloned in pACYC177. Deletion of *dgoR* leads to faster growth of BW in D-galactonate, and this phenotype is complemented by WT DgoR expressed from the plasmid (22) (Fig. 1B through D). The ∆*dgoR* strain expressing the variants L3F, K15R, H19Q, A128S, T180S, A186V, S220C, and T222I exhibited growth similar to cells expressing WT DgoR (Fig. 1B). However, ∆*dgoR* expressing P24L and A152E variants displayed faster growth (Fig. 1C), while ∆*dgoR* expressing R71C and P92L variants exhibited slow and very slow growth, respectively, compared to the strain expressing WT DgoR (Fig. 1D). This is evident from the less time taken by ∆*dgoR* expressing P24L and A152E variants to enter the exponential phase than ∆*dgoR* expressing WT protein (WT: ~14 hours, P24L: ~6 hours, and A152E: ~7 hours; Fig. 1C) and the higher doubling time of ∆*dgoR* expressing R71C and P92L variants than ∆*dgoR* expressing WT DgoR (WT: 1.62 ± 0.05 hours, R71C: 2.88 ± 0.49 hours, and P92L: 4.86 ± 0.52 hours; Fig. 1D). Residue P24 is located in the DNA-binding domain, R71 is located in the linker, and P92 and A152 are located in the E-O domain of DgoR (Fig. 1E).

**Fig 2:**
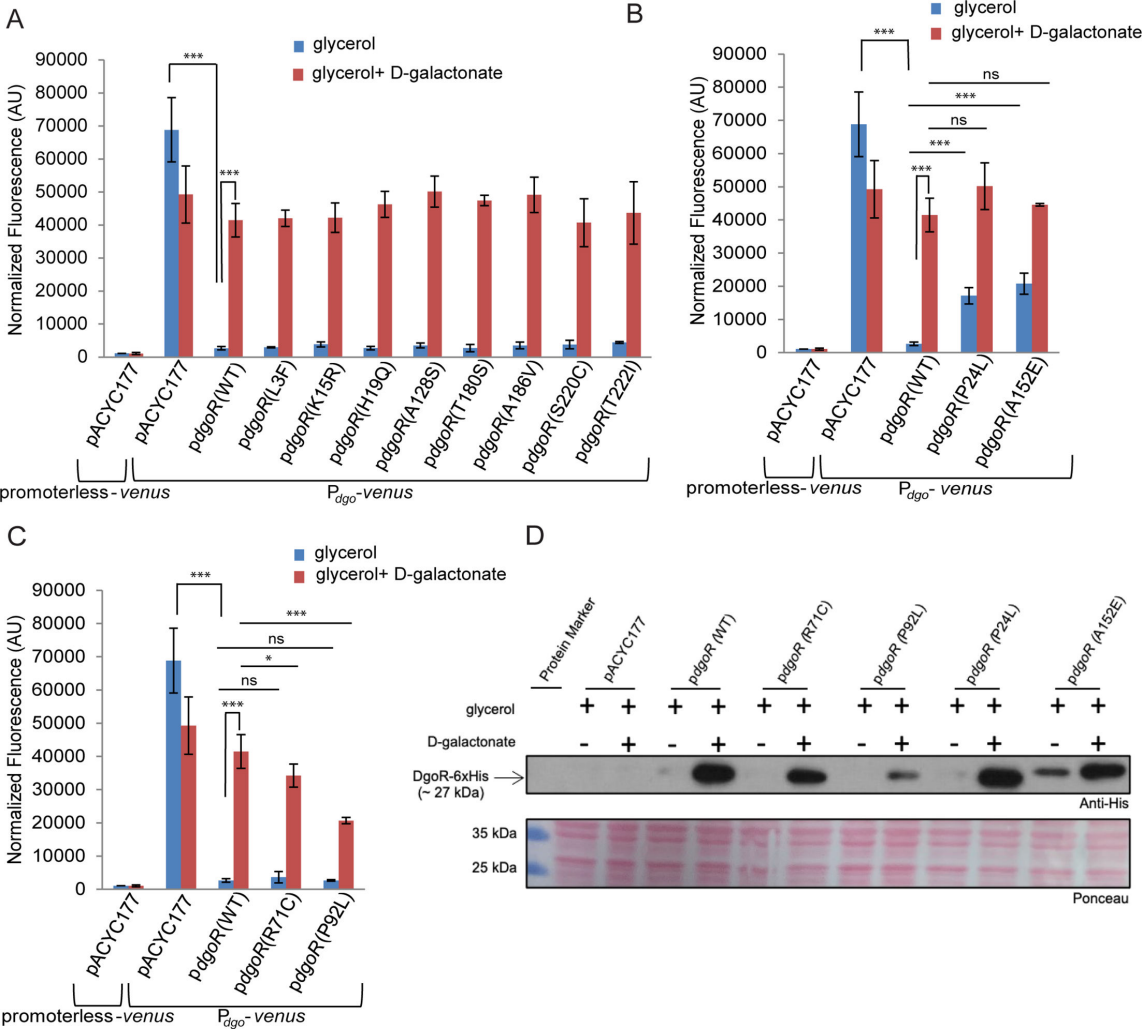
Of the 12 unique amino acid changes in DgoR among the natural isolates, four variations either compromise the repression ability or decrease the response to D-galactonate. (**A–C**) P24L and A152E variants are compromised for repression, while R71C and P92L variants are less responsive to D-galactonate. The BW Δ*dgoR* strain carrying the fluorescent Venus reporter on the chromosome under the control of the BW *dgo* promoter was transformed either with the plasmid pACYC177 or the p*dgoR* clones mentioned in Fig. 1B through D. The BW Δ*dgoR* strain carrying promoterless Venus on the chromosome and plasmid pACYC177 was used as a control for background fluorescence. Strains were grown in minimal medium containing either glycerol (non-inducing medium) or glycerol and D-galactonate (inducing medium). Fluorescence was measured and normalized to the OD_450_ of the samples. Data represent the average (±SD) from at least three independent experiments. (**A**) DgoR variants that behave similarly to WT in both repressing the *dgo* promoter in the non-inducing medium and responding to D-galactonate in the inducing medium. (**B**) DgoR variants that enable partial constitutive expression from the *dgo* promoter. (**C**) DgoR variants defective in responding to D-galactonate. Data for all the strains shown in panels A–C were generated together; hence, the controls in these figures, i.e., data for promoterless-*venus* (pACYC177), P*_dgo_-venus* (pACYC177), and P*_dgo_-venus* (p*dgoR* WT), are the same. (**D**) The expression of WT DgoR and its variants from the *dgo* promoter correlates with the repression ability of the proteins. The strains mentioned in Fig. 1C and D were grown in a minimal medium containing either glycerol or glycerol and D-galactonate. Cells were harvested in the exponential phase and processed for Western blotting. The blot was probed with an anti-His antibody. Ponceau S-stained counterpart of the Western blot was used as a loading control. The blot shown is representative of two independent replicates. For panels A–C, the *P*-values were calculated using the unpaired two-tailed Student’s *t* test (***P < 0.001; **P < 0.01; *P < 0.03; and ns, *P* > 0.03). For panel A, the reporter expression for each variant compared to the reporter expression for WT, either in non-inducing or inducing medium, did not show a significant difference.

Using transcriptional reporter assays, we next investigated whether DgoR variants have an altered ability to repress the *dgo* operon or altered sensitivity to D-galactonate. For this, WT DgoR or its variants were expressed from pACYC177 in a ∆*dgoR* strain where the fluorescent Venus reporter transcriptionally fused to the *dgo* promoter was integrated at the *att*λ site. Using this reporter strain, we have previously shown that the regulation of Venus expression by DgoR mimics the regulation of the *dgo* operon by the repressor (22). Whereas WT DgoR represses Venus expression in a non-inducing medium (minimal medium containing glycerol), Venus expression is considerably higher in an inducing medium (minimal medium containing glycerol and D-galactonate) (Fig. 2A through C). Consistent with growth in D-galactonate, except P24L, R71C, P92L, and A152E, all other variants behaved similarly to WT in both repressing the *dgo* promoter as well as responding to D-galactonate (compare Fig. 1B and 2A). The P24L and A152E variants enabled similar reporter expression as the WT DgoR in the inducing medium; however, they allowed partial constitutive expression of the reporter in the non-inducing medium (Fig. 2B). The partial constitutive expression of *dgo* genes in the ∆*dgoR* strain expressing these variants likely explains their faster growth in D-galactonate (compare Fig. 1C and 2B). Furthermore, while R71C and P92L variants behaved similarly to WT in non-inducing medium, they were induced to a lesser extent by D-galactonate, which likely explains their slower growth in D-galactonate (compare Fig. 1D and 2C).

**Fig 3:**
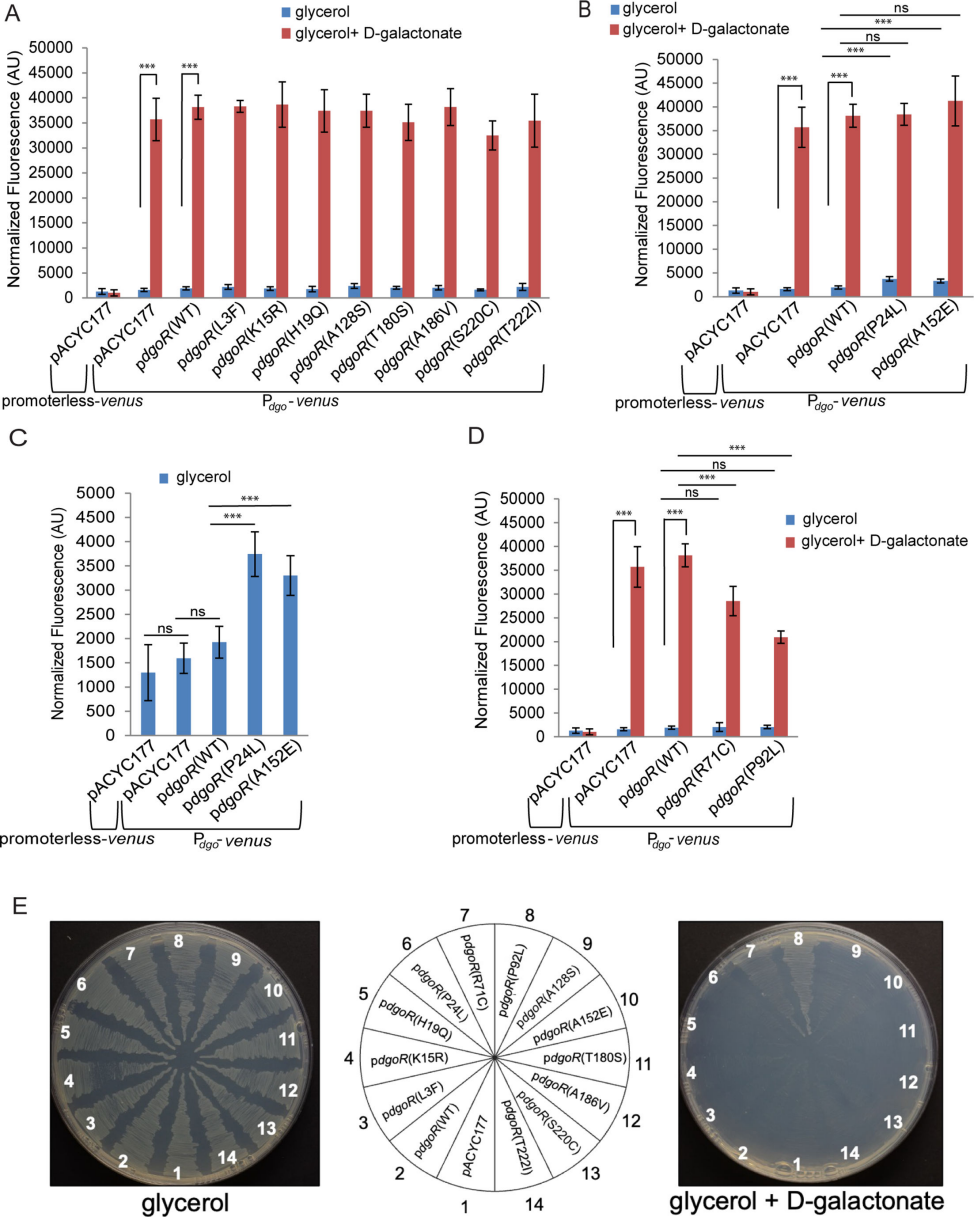
P24L, R71C, P92L, and A152E variants show genetic dominance over WT DgoR. (**A through D**) The BW WT strain carrying the fluorescent Venus reporter on the chromosome under the control of the BW *dgo* promoter was transformed either with plasmid pACYC177 or the p*dgoR* clones described in Fig. 2. The BW WT strain carrying promoterless Venus on the chromosome and plasmid pACYC177 was used as a control for background fluorescence. Growth and fluorescence measurement conditions were similar as described in the legend to Fig. 2A to C. Data represent the average (±SD) from at least four independent experiments. (**A**) DgoR variants that behave similarly to WT in both non-inducing and inducing media. (**B and C**) DgoR variants that exhibit a dominant negative phenotype. Because the normalized fluorescence values for strains grown in the non-inducing medium are considerably lower than those grown in the inducing medium, the data for strains grown in the non-inducing medium from panel B have been re-plotted in panel C for better visualization. (**D**) DgoR variants that behave as superrepressors. Data for all the strains shown in panels A–D were generated together; hence, the controls, i.e., data for promoterless-*venus* (pACYC177), P*_dgo_-venus* (pACYC177), and P*_dgo_-venus* (p*dgoR* WT) are the same. (**E**) The BW ∆*dgoA* strain expressing R71C and P92L variants can grow on a medium containing glycerol and D-galactonate. The BW ∆*dgoA* strain was individually transformed with the plasmid pACYC177 and p*dgoR* clones mentioned in Fig. 1. The transformants were streaked on M9 minimal medium containing either glycerol or glycerol and D-galactonate. Minimal medium supplemented with glycerol was used as a control, where all the strains grew normally. The experiment was done three times. A representative image is shown. For panels A–D, the *P*-values were calculated using the unpaired two-tailed Student’s *t* test (***P < 0.001; **P < 0.01; *P < 0.03; and ns, *P* > 0.03). For panel A, the reporter expression for each variant compared to the reporter expression for WT, either in non-inducing or inducing medium, did not show a significant difference.

Because DgoR is an auto-repressor, the expression of WT and mutant proteins from the *dgo* promoter should correlate with their repression ability (22, 26, 28). We thus compared the level of the four DgoR variants that showed altered behavior in fluorescence reporter assays with that of the WT protein expressed from the *dgo* promoter in the ∆*dgoR* strain in both non-inducing and inducing media. As expected, whereas in the non-inducing medium WT DgoR was not detected due to autorepression, it was considerably expressed in the inducing medium due to relief of repression by D-galactonate (Fig. 2D). Corroborating with the reporter data, in the inducing medium, P24L and A152E variants were expressed to a similar level as WT protein; however, in the non-inducing medium, the expression of A152E was higher than WT protein, suggesting that the variant is compromised in repression ability (Fig. 2D). Whereas the fluorescent reporter data suggested a slight loss of repression ability of the P24L variant (Fig. 2B), the protein could not be detected in the non-inducing medium (Fig. 2D), likely due to its rapid degradation. However, the low-level expression of P24L protein inside the cell is evident from its ability to exhibit a dominant negative phenotype (see below [Fig. 3B and C]). Consistent with the reporter data, in the non-inducing medium, R71C and P92L variants were not detected due to autorepression; however, these proteins were detected in the inducing medium but to a level lower than WT DgoR, with P92L showing the lowest expression (Fig. 2D), reiterating that these variants are less responsive to D-galactonate compared to the WT protein.

Collectively, the expression of the fluorescent reporter and DgoR from the *dgo* promoter indicates that P24L and A152E variations compromise the repression ability of DgoR, whereas R71C and P92L variations decrease the response of the repressor to D-galactonate.

### DgoR variants with compromised repression ability or reduced effector sensitivity are dominant over the wild-type repressor

Because DgoR forms dimers, its DNA-binding-defective mutants expressed in *trans* oligomerize with WT DgoR from the chromosome, interfering with its binding to the *dgo* promoter, thereby exhibiting a dominant-negative phenotype (22). Furthermore, DgoR mutants with reduced effector sensitivity behave as superrepressors (26, 28). As an additional assay to assess which amino acid changes in DgoR among the natural variations result in altered repression ability or sensitivity to D-galactonate, we tested the DgoR variants for genetic dominance. For this, we expressed WT DgoR or its variants from the *dgo* promoter (from pACYC177) in the WT strain carrying fluorescent Venus reporter on the chromosome under the control of the *dgo* promoter (WT reporter strain) (22). In the non-inducing as well as the inducing medium, cells expressing variants L3F, K15R, H19Q, A128S, T180S, A186V, S220C, and T222I from the plasmid and WT DgoR from the chromosome showed similar reporter expression as the cells expressing WT DgoR both from the plasmid and the chromosome, indicating that these eight variants do not affect the repressor (Fig. 3A). However, compared to the strain expressing WT DgoR both from the plasmid and the chromosome, in the non-inducing medium, strains expressing either P24L or A152E variants from the plasmid and WT DgoR from the chromosome showed increased reporter expression (Fig. 3B and C), whereas, in the inducing medium, strains expressing either R71C or P92L variants from the plasmid and WT DgoR from the chromosome showed decreased expression of the reporter (Fig. 3D). Collectively, the dominant negative behavior of P24L and A152E variants and the superrepressor phenotype of R71C and P92L variants emphasize that these mutants have decreased repression ability and are less responsive to D-galactonate, respectively.

We previously exploited the phenotype of the ∆*dgoA* strain to establish the superrepressor phenotype of DgoR mutants corresponding to amino acid residues in the E-O domain (26, 28). Here, the ∆*dgoA* strain expressing WT DgoR is unable to grow on a medium containing glycerol and D-galactonate because the absence of aldolase, DgoA, results in the accumulation of a toxic phosphorylated intermediate (2-dehydro-3-deoxy-D-galactonate 6-phosphate). On the other hand, mutants defective in responding to D-galactonate grow on this medium because of the compromised derepression of the D-galactonate metabolic pathway, and hence, no/less accumulation of the toxic metabolite (26, 28, 47). To further validate that among the 12 unique amino acid changes in DgoR among the natural isolates, only R71C and P92L exhibit superrepressor phenotype, we individually expressed WT DgoR or its 12 variants from the *dgo* promoter (from pACYC177) in the ∆*dgoA* strain (harboring the chromosomal copy of WT *dgoR*) and streaked the resulting transformants on minimal medium supplemented with glycerol and D-galactonate. Indeed, we only observed the growth of the ∆*dgoA* strain expressing R71C or P92L variants (Fig. 3E). These results corroborate with data from other *in vivo* assays (Fig. 1D; 2C and D; 3D).

### A152E and R71C variations impact the growth of natural isolates in D-galactonate

**Fig 4:**
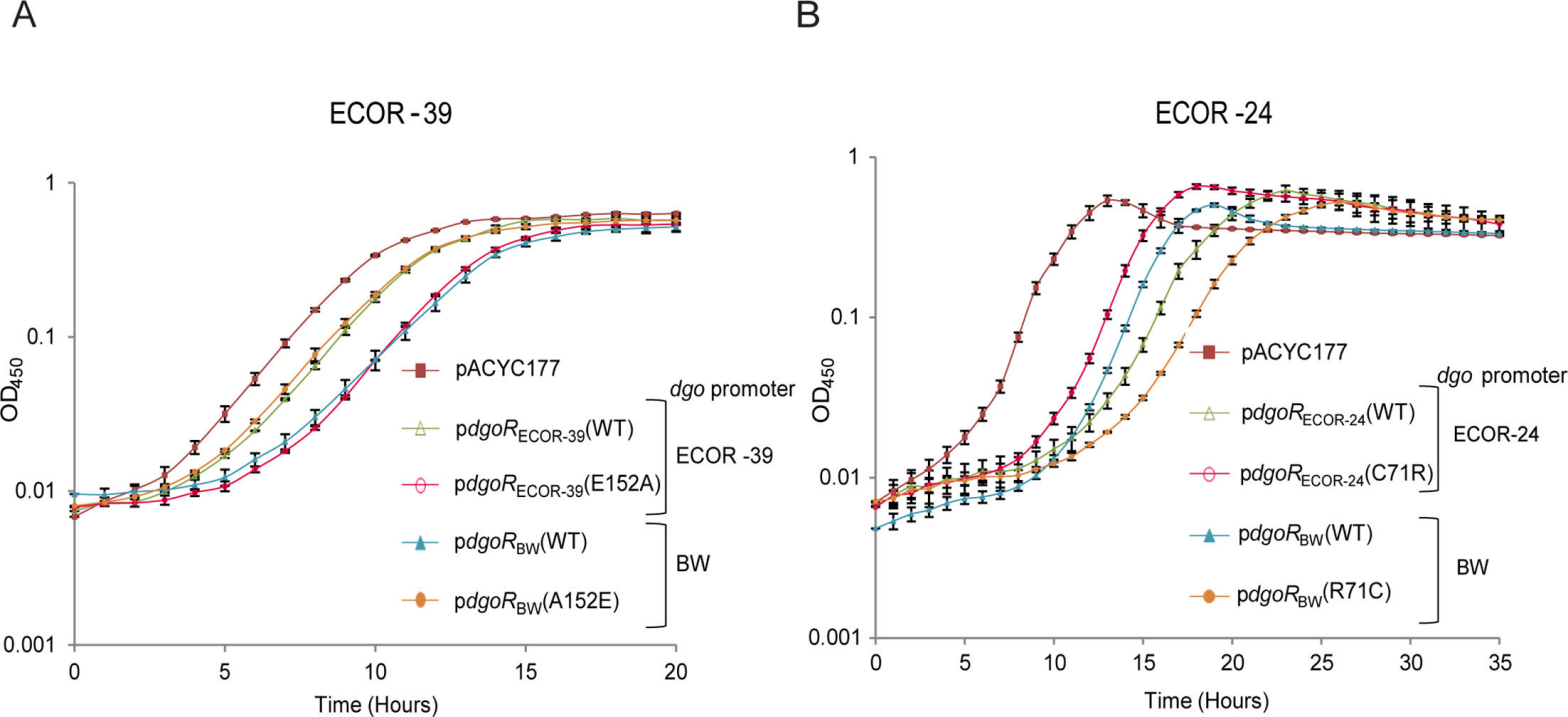
A152E variation in DgoR leads to the faster growth of ECOR-39, while R71C variation in DgoR leads to the slower growth of ECOR-24 in D-galactonate. (**A**) A152E variation in DgoR results in the faster growth of ECOR-39 in D-galactonate. The plasmid pACYC177, pACYC177 carrying BW *dgoR* cloned under BW *dgo* promoter [p*dgoR*_BW_(WT), pBS13 and p*dgoR*_BW_(A152E), pSW26] and pACYC177 carrying ECOR-39 *dgoR* cloned under ECOR-39 *dgo* promoter [p*dgoR*_ECOR-39_(WT), pSW24 and p*dgoR*_ECOR-39_(E152A), pSW25] were individually transformed in two parental backgrounds of the ECOR-39 *dgoR::kan* strain (Parents 1 and 2). Cultures were grown in an M9 minimal medium supplemented with D-galactonate as the sole carbon source, and OD_450_ was measured. The experiment was performed two times, each with transformants of Parents 1 and 2 (Fig. S2); each experiment had three technical replicates. A representative data set, with average (±SD) from technical replicates, for transformants of Parent 1 is shown. (**B**) R71C variation in DgoR results in a slower growth of ECOR-24 in D-galactonate. The plasmid pACYC177, pACYC177 carrying BW *dgoR* cloned under BW *dgo* promoter [p*dgoR*_BW_(WT), pBS13 and p*dgoR*_BW_(R71C), pSW28], and pACYC177 carrying ECOR-24 *dgoR* cloned under ECOR-24 *dgo* promoter [p*dgoR*_ECOR-24_(WT), pSW35 and p*dgoR*_ECOR-24_(C71R), pSW38] were individually transformed in two parental backgrounds of the ECOR-24 *dgoR::kan* strain (Parents 1 and 2). Cultures were grown in an M9 minimal medium supplemented with D-galactonate as the sole carbon source, and OD_450_ was measured. The experiment was performed two times, each with transformants of Parents 1 and 2 (Fig. S3); each experiment had three technical replicates. A representative data set, with average (±SD) from technical replicates, for transformants of Parent 1 is shown. Because P1 transduction was unsuccessful in ECOR-24 and ECOR-39 strains, we performed the above experiments in two parental backgrounds of both strains to validate the phenotypes.

The variations P24L and A152E, which are naturally present in the *E. coli* isolates, P24L in UTI 83972 and HM-50, and A152E in ECOR-39 and ECOR-40 (46) (Table S1), decrease the repressor ability of BW DgoR (Fig. 1C, 2B, D, 3B, and C). On the other hand, the variations R71C and P92L, which are naturally present in the *E. coli* isolates, R71C in ECOR-24 and IAI13, and P92L in NILS 56 (46) (Table S1), decrease the response of BW DgoR to D-galactonate (Fig. 1D, 2C, D, 3D, and E). Here, we investigated the contribution of one of the amino acid variations that decrease the repression ability (A152E) or decrease the response to the effector (R71C) on the growth of natural isolates in D-galactonate. For this, we deleted the chromosomal copy of *dgoR* in gut commensals, ECOR-39 and ECOR-24. We then complemented (i) ECOR-39 ∆*dgoR* strain with pACYC177 expressing (a) BW WT DgoR (harboring A152) and BW A152E DgoR from BW *dgo* promoter, and (b) ECOR-39 WT DgoR (harboring A152E variation) and ECOR-39 E152A DgoR from ECOR-39 *dgo* promoter, and (ii) ECOR-24 ∆*dgoR* strain with pACYC177 expressing (a) BW WT DgoR (harboring R71) and BW R71C DgoR from BW *dgo* promoter, and (b) ECOR-24 WT DgoR (harboring R71C variation) and ECOR-24 C71R DgoR from ECOR-24 *dgo* promoter. The expression of BW WT DgoR results in a slower growth of the ECOR-39 ∆*dgoR* strain compared to the expression of BW A152E DgoR. Furthermore, ECOR-39 ∆*dgoR* expressing ECOR-39 WT DgoR shows faster growth than the strain expressing ECOR-39 E152A DgoR. This is evident from the time taken by the ECOR-39 ∆*dgoR* strain expressing the various DgoR proteins to enter the exponential phase (BW WT DgoR: ~5.5 hours, BW A152E DgoR: ~4 hours, ECOR-39 WT DgoR: ~4 hours, and ECOR-39 E152A DgoR: ~6 hours) (Fig. 4A; Fig. S2). For the ECOR-24 ∆*dgoR* strain, the expression of BW WT DgoR results in faster growth compared to BW R71C DgoR. On the other hand, the expression of ECOR-24 WT DgoR leads to a slower growth of ECOR-24 ∆*dgoR* compared to ECOR-24 C71R DgoR. This is evident from the doubling time of the ECOR-24 ∆*dgoR* strain expressing the various DgoR proteins (BW WT DgoR: 1.23 ± 0.01 hours, BW R71C DgoR: 1.81 ± 0.04 hours, ECOR-24 WT DgoR: 1.67 ± 0.04 hours, and ECOR-24 C71R DgoR: 1.31 ± 0.03 hours) (Fig. 4B; Fig. S3). Collectively, these data validate that, similar to the laboratory *E. coli* strain (BW), amino acid variations in DgoR also impact the growth of natural isolates in D-galactonate.

### The A152E variant has reduced affinity for the *dgo* promoter, while the R71C variant releases the promoter at a higher effector concentration

**Fig 5:**
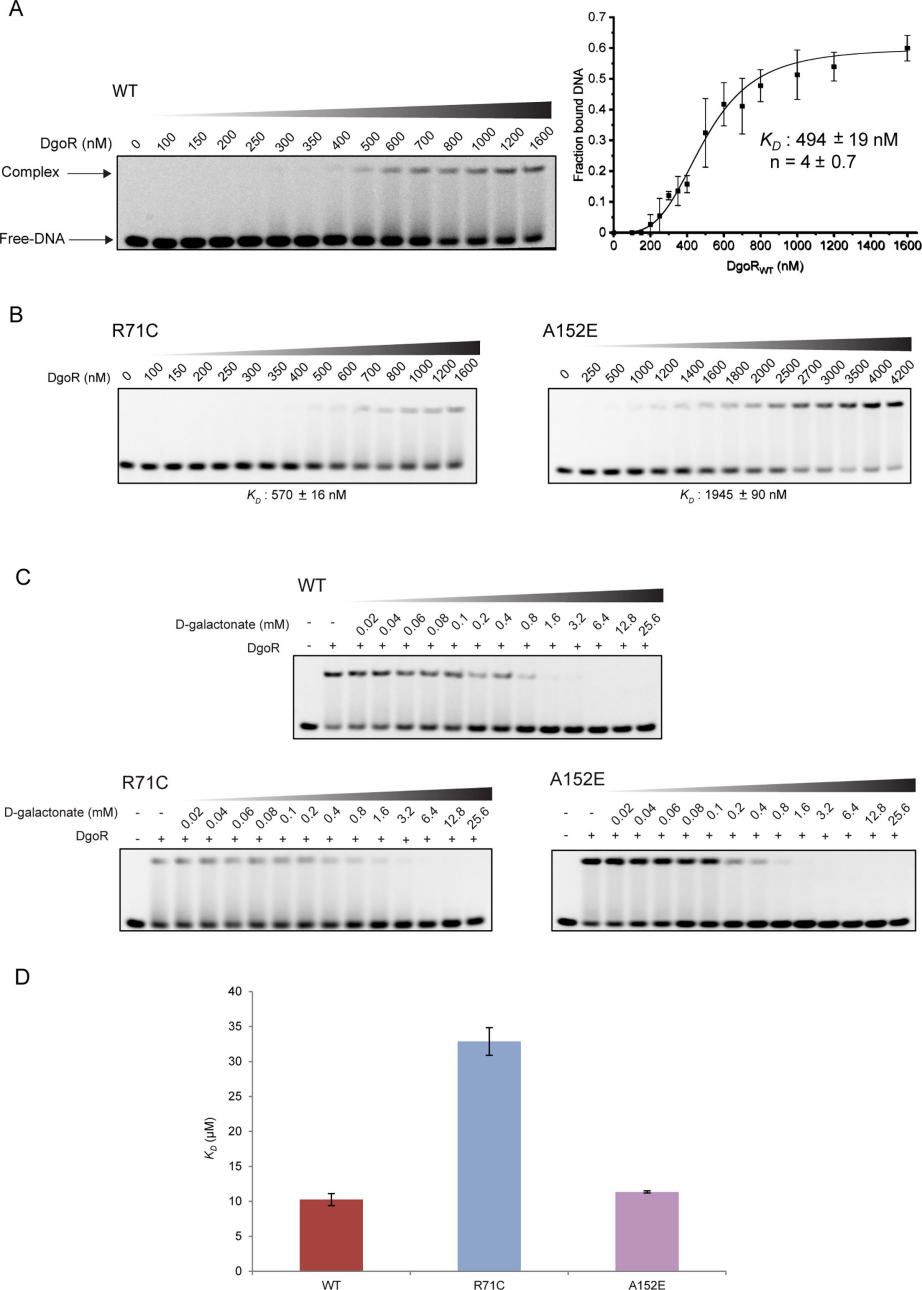
The A152E variant binds the *dgo* promoter with lower affinity, while the R71C variant releases the promoter at a higher D-galactonate concentration. (**A**) The apparent affinity constant (*K_D_*) for the interaction of BW WT DgoR with the *dgo* promoter. Left: the 5′-end Cy5-labeled DNA probe (10 nM) was incubated with increasing concentrations of purified WT DgoR-6XHis for 30 min, and the samples were resolved on native PAGE gels. Right: bands corresponding to free DNA and protein-DNA complex were quantified to determine the apparent *K_D_*. Each data point in the plot represents the average (±SD) of three independent experiments. “n” is the Hill coefficient. (**B**) The apparent *K_D_* for the interaction of DgoR variants with the *dgo* promoter. The labeled probe was incubated with increasing concentrations of the purified DgoR variants, and apparent *K_D_* was measured as described in panel A. (**C**) The R71C variant requires a higher D-galactonate concentration to release the promoter. Purified BW WT or variant DgoR-6XHis (1 µM for WT and R71C; 3.5 µM for A152E) was incubated with increasing concentrations of D-galactonate, as indicated, for 20 min. The 5′-end Cy5-labeled DNA probe (10 nM) was added, and the samples were further incubated for 30 min. The samples were resolved on native PAGE gels. The experiment was performed three times. A representative data set is shown. (**D**) R71C variant has a weaker affinity for D-galactonate. Serially diluted D-galactonate (100 µM –3.05 nM) and BW WT DgoR-6XHis or its variants (500 nM) were incubated for 15 min at room temperature. The samples were then loaded into label-free capillaries. Binding assays were conducted, and data were analyzed and plotted as described in Materials and Methods. The data represent the average *K_D_* values (±SD) from three independent experiments. The representative MST plots are shown in Fig. S5.

We next wanted to investigate whether the slight loss of repression of the *dgo* operon in cells expressing P24L and A152E variants was because of the lower affinity of these mutants for the *dgo* promoter and whether the superrepressor behavior of R71C and P92L variants was due to their increased affinity for the *dgo* promoter or decreased response to D-galactonate. We thus attempted purification of all four DgoR variants along with BW WT DgoR as C-terminally His-tagged proteins from isopropyl-α-D-thiogalactopyranoside (IPTG)-inducible P*_trc_* promoter. The overexpression of the P24L variant could not be achieved upon IPTG induction, and although the P92L variant was overexpressed upon induction, it could not be purified to homogeneity. We, therefore, proceeded with the *in vitro* analyses of the R71C and A152E variants. Far-UV circular dichroism and size exclusion chromatography showed that these variants are folded and elute at a retention volume similar to the WT protein, respectively (Fig. S4). The electrophoretic mobility shift assays (EMSAs) showed that WT DgoR and R71C variant bind the promoter with similar affinity (WT, *K_D_* = 494 ± 19 nM and R71C, *K_D_* = 570 ± 16 nM) (Fig. 5A and B); however, A152E variant binds the promoter with approximately fourfold weaker affinity (*K_D_* = 1,945 ± 90 nM; Fig. 5B). Furthermore, whereas D-galactonate completely abrogated the binding of WT DgoR and the A152E variant to the *dgo* promoter at a concentration of 1.6 mM, 6.4 mM D-galactonate was required to completely release the *dgo* promoter bound to R71C (Fig. 5C). Next, we performed microscale thermophoresis (MST) to examine if the slightly weak response of R71C to D-galactonate is because of its reduced affinity for the effector. Whereas WT and A152E had a comparable affinity for D-galactonate (WT, *K_D_* = 10.3 ± 0.3 µM and A152E, *K_D_* = 11.3 ± 0.2 µM), R71C had approximately threefold weaker affinity for the effector (*K_D_* = 32.9 ± 1.2 µM) (Fig. 5D; Fig. S5). Taken together, the *in vitro* analyses support the conclusion that the partial loss of repression observed for the A152E variant *in vivo* is due to its decreased affinity for the *dgo* promoter, and the superrepressor behavior of the R71C variant is due to its reduced ability to respond to D-galactonate.

### Variations affect the essential dynamics and alter the dynamic network involved in allosteric communication

Because R71C located in the linker region weakens effector binding and A152E present in the E-O domain weakens DNA binding, we used MD simulations to probe their effect on allosteric communication in DgoR. Previously, we proposed a potential mechanism for allosteric communication regulating the function of WT DgoR in the presence of its effector and DNA (28). Four predominant conformational states are available for DgoR: APO state (a functional dimeric form of DgoR), E-bound state (effector-bound state where effector molecules bind to the C-terminal domains of DgoR), D-bound state (DNA-bound state where DgoR binds to DNA via its N-terminal domains), and ED-bound state (where the effector binds to the C-terminal domains and DNA binds to the N-terminal domains). MD simulations suggested that the N-terminal and C-terminal domains allosterically modulate DgoR activity by altering its conformational dynamics in response to DNA interaction and effector binding. To understand how the natural isolate variations influence the functioning of DgoR, we carried out MD simulations of variants in four states, APO, E-bound, D-bound, and ED-bound, and compared these with the simulations for WT.

**Fig 6:**
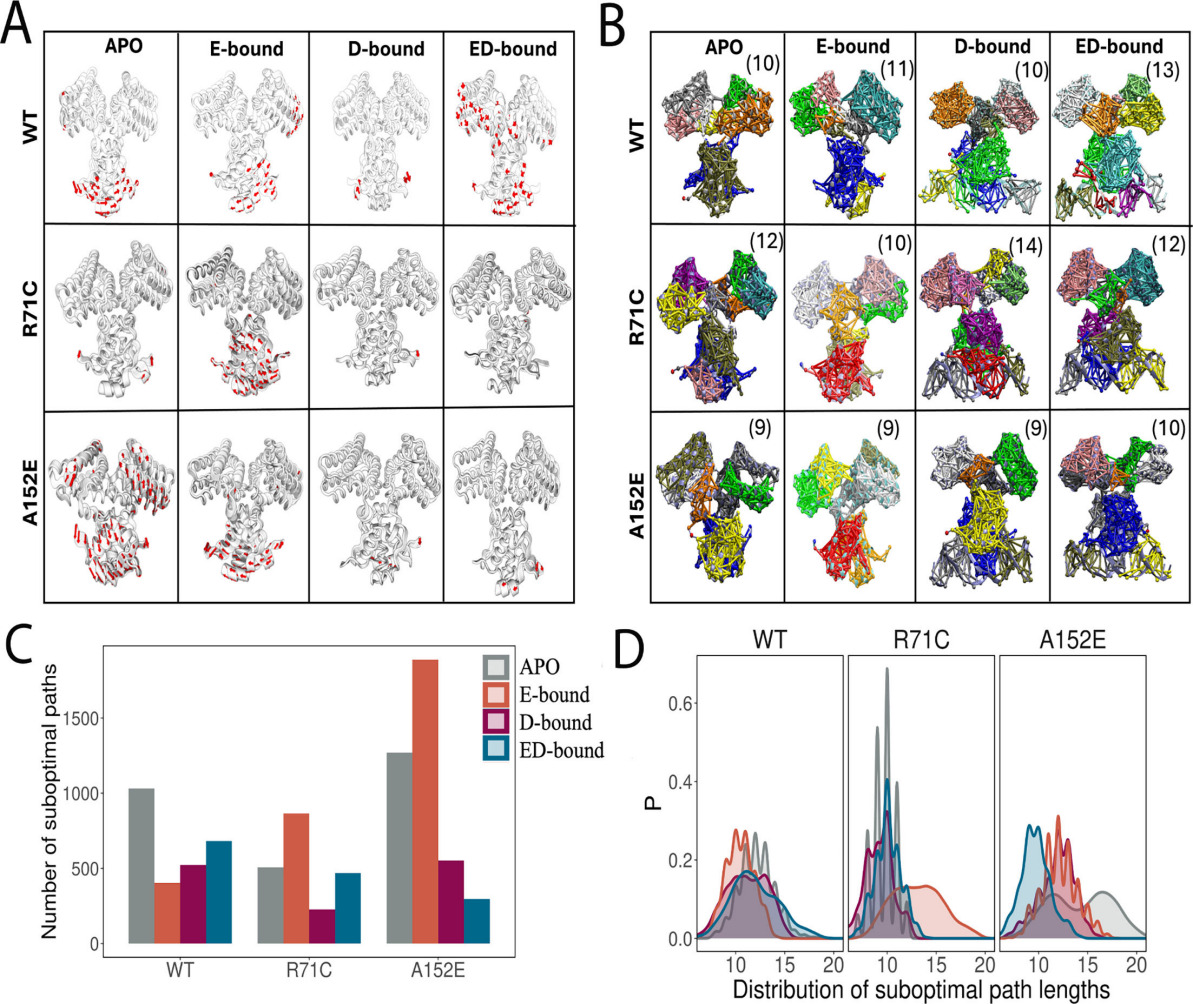
(**A**) Motion along the top principal component (PC1) for different states of WT and variant DgoR complexes. Red arrows denote the direction and magnitude of displacement with cutoff values of 3.5 Å. (**B**) Networks split into distinct communities, each highlighted by a different color, are shown for the four states of WT and variant DgoR complexes. The values in parentheses indicate the total number of communities observed for each respective state. (**C**) Total number of suboptimal paths between the N-terminal and C-terminal regions for WT and variant DgoR complexes across different states. (**D**) Distribution of suboptimal path lengths computed for allosteric communication between the N-terminal and C-terminal domains of WT and variant DgoR complexes in different states.

We used root mean square deviation and root mean square fluctuation analyses to compare structural stabilities of WT DgoR and its variants across different simulated states (Fig. S6). In general, higher deviations and fluctuations in APO, E-bound, and ED-bound states indicate conformational flexibility, particularly in the C-terminal domain. To assess how allosteric signals from DNA binding (N-terminal) or effector binding (C-terminal) couple domain motions within DgoR, we computed dynamic cross-correlation (DCC) coefficients for each Cα atom and generated DCC maps for the WT and its variants (Fig. S7). DCC maps for WT revealed that effector binding at the C-terminal domain induces allosteric modulation of the N-terminal DNA-binding domain. Variant-specific correlation patterns highlight altered allosteric communication. Notably, R71C impairs DNA release due to rigid correlations, and while A152E shows disrupted DNA interaction (DNA binding does not induce effective synchronized motions), it shows WT-like response to the effector. We also analyzed the long-range correlated motions in the topmost principal component (PC) for WT and variants (Fig. 6A). MD simulations along PC1 revealed allosteric domain motions in WT and variant DgoR across different states. WT showed significant N-terminal motions in APO- and ED-bound states and synchronized domain movement in the E-bound state, indicating allosteric regulation. Compared to WT, in APO- and ED-bound states, R71C displayed reduced motion, suggesting impaired DNA release, while A152E retained flexibility and WT-like behavior. Overall, effector binding appears to facilitate DNA release in WT and A152E but not in the R71C variant due to restricted dynamic coupling. Next, we performed dynamical network analysis to observe communication pathways by integrating MD-derived correlation data with structure-based residue communities (Fig. 6B). WT DgoR showed state-dependent community reorganization, with increased complexity in the ED-bound state due to effector and DNA interactions. The variant R71C exhibited distinct patterns, indicating altered domain coupling and potential impacts on DNA release. To assess how variations affect allosteric communication, we computed the number (Fig. 6C) and length distributions (Fig. 6D) of suboptimal paths across different states of WT and DgoR variants. While more suboptimal paths may offer alternative communication routes, longer path lengths (right-shifted distributions) indicate delayed signal transmission, whereas shorter path lengths (left-shifted distributions) suggest faster communication. In WT DgoR, allosteric communication consistently involved both monomers, with the highest number of suboptimal paths in the APO state, which dropped in the E- and D-bound states but increased again in the ED-bound state, indicating effector-induced alternate signaling routes (Fig. 6C). Despite fluctuations in path numbers, path length distributions remained stable, ensuring efficient communication (Fig. 6D). Although compared to WT, the E-bound state of R71C showed an increase in the number of suboptimal paths (Fig. 6C), a right-shifted distribution suggests hindered effector signaling (Fig. 6D). Furthermore, although in the D-bound state of A152E, the number of suboptimal paths remained similar to WT (Fig. 6C), a right-shifted distribution indicates disfavored DNA binding (Fig. 6D). For a detailed explanation of these simulation results, please refer to the supplemental material.

## DISCUSSION

### Integrating experimental findings with simulation data elucidates the functional consequences of genetic variations in *dgoR*

Using a combination of genetics, biochemical assays, and MD simulations, this study provides insights into the dynamics and functional outcome of amino acid variations in DgoR, a key regulator of the *dgo* operon. The experimental data on the effect of 12 unique variations in DgoR, present among 340 sequenced natural isolates, on the growth of a laboratory strain of *E. coli* in D-galactonate, expression of Venus reporter and DgoR from the autoregulated *dgo* promoter, and genetic dominance validated the compromised repression ability of P24L and A152E, and the reduced sensitivity of R71C and P92L to D-galactonate (Fig. 1 to 3). *In vitro* experiments confirmed that the compromised inducibility of R71C is due to its reduced response to the effector, and the attenuated repression ability of A152E is due to its decreased affinity for the *dgo* promoter (Fig. 5). Extending the analysis to ECOR-39 (harboring A152E variation) and ECOR-24 (harboring R71C variation) revealed that amino acid variations indeed determine the growth of natural isolates in D-galactonate (Fig. 4).

**Fig 7:**
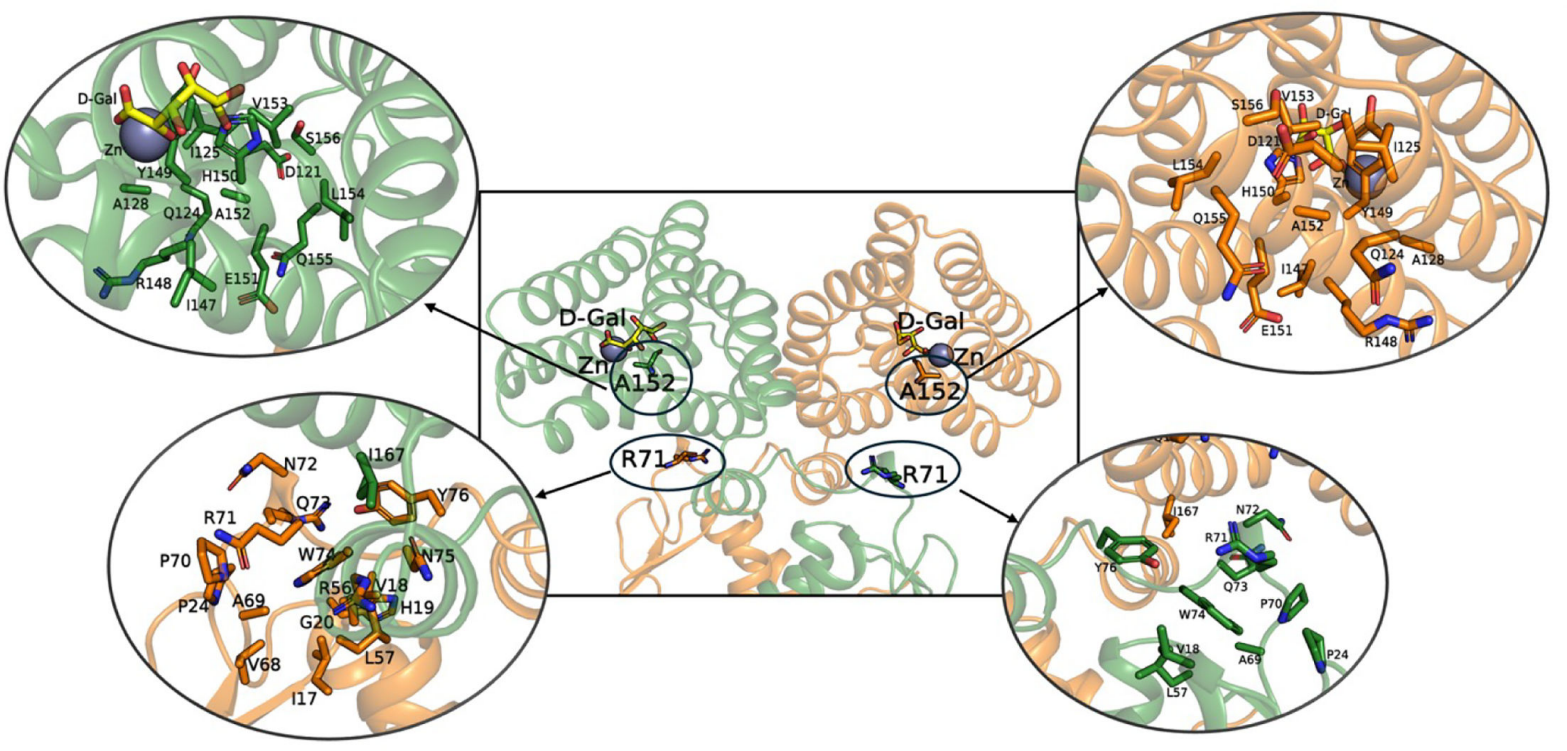
Detailed interactions of variation sites in DgoR. D-galactonate (D-Gal) and Zn ion are shown in yellow sticks and a purple sphere, respectively. Residues in Monomer 1 are shown in green-colored sticks and ribbon, and residues in Monomer 2 are shown in orange-colored sticks and ribbon. Main chain atoms are not shown for clarity.

R71 is positioned in the linker region; however, its mutation decreases the effector-binding affinity. On the other hand, the mutation of A152, located in the E-O domain, reduces the DNA-binding affinity. Locating the mutation sites in the modeled structure of the DgoR dimer showed that R71 interacts with I167 in the E-O domain (Fig. 7). Mutating R71 likely disrupts its interaction with I167, thereby reducing effector binding to DgoR. A152 is in proximity to E151, which plays a crucial role in allosteric communication by interacting with the linker region of the adjacent monomer via N72 (Fig. 7). We recently showed that the E151A mutation renders DgoR completely DNA-binding defective (28). Thus, the A152E mutation likely influences DNA binding due to altered interactions associated with E151. Extensive MD simulations indeed revealed that the correlation patterns, dynamics, and networks of R71C and A152E variants in response to DNA and effector binding are distinct from WT (Fig. 6; Fig. S6 and S7).

### Possible physiological consequences of genetic variations in *dgoR*

The *dgo* operon is present in a large number of enterobacterial strains, indicating the widespread use of D-galactonate as a carbon source (46, 48, 49). These strains include both commensals and pathogens isolated from different host niches, including blood, urine, and gut, environments where the presence of D-galactonate has been reported (2, 5, 6, 50–53). In long-term evolution experiments where *E. coli* isolates were adapted to the mouse gut, the adaptive mutations targeting *dgoR* often involved IS insertions, indels, or nonsense mutations that likely inactivate the repressor and thus would result in the overexpression of the *dgo* operon. Notably, *dgoR* was mutated in invader *E. coli* only in the absence of the resident *E. coli* (38–40). These observations suggest that D-galactonate is a limiting nutrient inside the mammalian gut and that natural isolates harboring variations in *dgoR* that either increase the sensitivity of the repressor to D-galactonate or result in constitutive expression of the *dgo* operon might gain a competitive edge over other members of the microbiota.

Single amino acid variations in TRs of carbon metabolism are known to dramatically impact the outcome of bacteria inside their hosts—in certain ribotypes of *Clostridium difficile*, a point mutation in *treR*, a transcriptional repressor of trehalose metabolism, increases their sensitivity to trehalose, enabling them to metabolize sugar at lower concentrations and attain hypervirulence (42); several outbreak isolates of *Enterococcus faecalis* harbor point mutations in *gntR*, a transcriptional repressor of polysaccharide metabolism, which confer these strains the ability to withstand antibiotic pressure and innate immune defenses in the human bloodstream (41); and a single amino acid change in RafR, a transcriptional activator of raffinose metabolism in *Streptococcus pneumoniae*, is associated with differential metabolism of raffinose among blood and ear isolates, which serves as an important determinant for their distinct tropism for lungs vs brain and ear in mice (43). Our study has identified four unique amino acid variations in DgoR that affect the growth of *E. coli* in D-galactonate. The aforementioned examples of amino acid variations in TRs underscore the importance of investigating the effect of variations in DgoR on bacterial colonization, virulence, and tropism using animal models. Interestingly, the asymptomatic bacteriuria *E. coli* strain 83972, which has significantly induced *dgo* genes when cultured in human urine and outcompetes uropathogenic *E. coli* strains in this niche (33), harbors the P24L variation in DgoR.

To explore the frequency of occurrence of genetic variations in DgoR that impacted its function (P24L, R71C, P92L, and A152E), we extrapolated different DgoR data sets beyond the panel of 340 sequenced natural *E. coli* isolates that we had considered (46). Alignment-based variation identification among the DgoR sequences across *E. coli* (323,578 strains) and order Enterobacterales (1,100,989 strains) revealed the presence of these variations; however, they are not very common (Dataset S1), suggesting that they may not have a significant role in the broader population. However, beyond the 12 variations studied here, this data set also revealed the presence of a large number of additional variations across *E. coli* and order Enterobacterales, highlighting the need to examine their impact on the DgoR function. Overall, these findings warrant further investigation into DgoR variations to characterize their functional roles and understand their potential physiological implications.

Several TRs of carbon metabolism also directly regulate virulence-associated genes. For instance, EHEC and *C. rodentium* use ExuR and PdhR, TRs of D-galacturonate degradation and central metabolism, respectively, as activators of the locus of enterocyte effacement (LEE) (30, 54), whereas they use FadR, a TR of long-chain fatty acid synthesis and degradation, to repress the expression of LEE-encoded genes (55). Notably, several *E. coli* isolates do not have the potential to metabolize D-galactonate because they either lack structural *dgo* genes or one or more structural *dgo* genes are a pseudogene; however, they still have intact *dgoR* (46). If similar to ExuR, PdhR, and FadR, DgoR also regulates virulence-related genes, then variations in *dgoR* might impact the virulence of these isolates independent of its role in metabolism.

Enteric bacteria must gauge their environment and adapt their metabolism and virulence strategies to establish themselves within the host. Our study has fundamental implications for how variations in TRs affect metabolic capabilities that eventually define the host range, the preferred niche of the organism, and colonization resistance by commensals or disease outcomes of pathogenic infections. Ultimately, this understanding could be leveraged to develop prebiotics and probiotics to combat infection and formulate drug development and therapeutic approaches.

## MATERIALS AND METHODS

### Media composition and culture conditions

The media with the following composition were used: lysogeny broth (LB), 5 g L^−1^ Bacto yeast extract, 10 g L^−1^ Bacto tryptone, and 5 g L^−1^ NaCl; and M9 minimal medium, 5.3 g L^−1^ Na_2_HPO_4_, 3 g L^−1^ KH_2_PO_4_, 0.5 g L^−1^ NaCl, 1 g L^−1^ NH_4_Cl, 0.12 g L^−1^ MgSO_4_, 2 mg L^−1^ biotin, 2 mg L^−1^ nicotinamide, 0.2 mg L^−1^ riboflavin, and 2 mg L^−1^ thiamine. The M9 minimal medium was supplemented with glycerol (0.4%, vol/vol), D-galactonate (10 mM, unless mentioned otherwise), or both glycerol and D-galactonate. Glycerol was obtained from Sigma. Calcium D-galactonate (MP Biomedicals) was used to prepare D-galactonate, as described (22). For solidifying media, Difco agar (1.5%, wt/vol) was used. Ampicillin, chloramphenicol, and kanamycin were used at a concentration of 100 µg mL^−1^, 12.5 µg mL^−1^, and 30 µg mL^−1^, respectively, when required. Overnight cultures set up in LB liquid medium (3 mL) were used to inoculate secondary cultures either in LB or in M9 minimal medium containing the desired carbon source with an initial optical density (OD) of ∼0.03. The cultures were incubated at 37°C, unless specified otherwise.

### Strains, plasmids, and primers

Tables S2 and S3 provide the list of bacterial strains and plasmids, and primers, respectively. *E. coli* DH5α was used for cloning in plasmids pACYC177 and pRC10. BL21(DE3) was used for protein expression and purification. BW25113 (designated as BW), ECOR-24, and ECOR-39 and deletion strains in these backgrounds were used for various *in vivo* assays.

For the fluorescence reporter assays, growth curves, Western blotting, and analysis of superrepressor phenotype using the BW ∆*dgoA* strain, BW WT DgoR and its various mutants were expressed as C-terminally 6XHis-tagged proteins from the BW *dgo* promoter using the plasmid pACYC177. Plasmid pBS13 was used to express BW WT DgoR (22). Overlap extension PCR was used to create *dgoR* variations in pACYC177 using pBS13 as the template. Plasmid pBS2 was used for overexpression and purification of C-terminally 6XHis-tagged BW DgoR (DgoR-6XHis) from the IPTG-inducible P*_trc_* promoter of plasmid pRC10 (22). For the expression and purification of BW DgoR variants, the mutated BW *dgoR* fragments were amplified from their constructs in pACYC177 and cloned into pRC10.

For fluorescence reporter assays, the strains RC12018 and RC12020, carrying a single copy transcriptional fusion of fluorescent Venus reporter with the BW *dgo* promoter in BW WT and ∆*dgoR* backgrounds, respectively, were used (22).

The strains RC16126 and RC16105, carrying a deletion of *dgoR* in ECOR-24 and ECOR-39, respectively, were constructed using the λ Red recombinase method (56). Briefly, the kanamycin cassette (flanked by regions homologous to the target site introduced through primers) was amplified from the plasmid pKD13 and transformed into ECOR-24 or ECOR-39 expressing λ Red recombinase enzymes from the pSIM5 helper plasmid. Recombinants were selected on LB-kanamycin plates. Strains were PCR verified. Because P1 transduction was unsuccessful in ECOR-24 and ECOR-39, for growth curve experiments, two parental colonies were used to ensure the reproducibility of phenotypes.

For growth curves that involved the expression of ECOR-24 and ECOR-39 DgoR, C-terminally 6XHis-tagged *dgoR* was cloned in pACYC177. The coding region of *dgoR*, along with its promoter (~200 bp upstream of the *dgoR* start codon), was PCR amplified from ECOR-24 and ECOR-39 and cloned into the *Aat*II and *Bam*HI sites of pACYC177 to generate plasmids pSW35 and pSW24, respectively. Furthermore, pSW35 and pSW24 were used as templates to create mutations in *dgoR* by overlap extension PCR, yielding plasmids pSW38 and pSW25, respectively.

### Growth curves

Overnight cultures grown in LB were pelleted, washed, and resuspended in M9 minimal medium without any carbon source. Secondary cultures were set up in 200 µL M9 minimal medium containing the desired carbon source to an initial OD_450_ of ∼0.03 in 96-well, clear bottom plates. Plates were placed in an incubator shaker, and OD_450_ of the cultures was recorded at regular intervals using a microplate reader (Tecan Infinite M200 monochromator). The reader and shaker were integrated with a liquid handling system (Tecan), allowing the automated transfer of plates between the two units.

### Fluorescence assays

Fluorescence assays were performed, as described previously (22). Briefly, overnight cultures of reporter strains grown in LB were harvested, washed, and resuspended in M9 minimal medium without any carbon source. Secondary cultures were grown with shaking in M9 minimal medium containing the desired carbon source, as described above for growth curve assays. However, here, the secondary cultures were grown in 96-well, black, clear-bottom plates (Costar, Corning). Fluorescence was measured in the exponential phase using a microplate reader (Tecan Infinite M200 monochromator) in the top mode with excitation and emission wavelengths of 498 and 568 nm, respectively. Fluorescence was normalized to OD_450_ and plotted in a bar graph.

### Western blotting

To monitor the expression of DgoR-6XHis, samples were electrophoresed on 15% SDS-PAGE gels and transferred to the nitrocellulose membrane. The membrane was blocked with skimmed milk (5%, wt/vol) at 4°C and probed with an anti-His primary antibody (1:1,000; Thermo Fisher Scientific) and horseradish peroxidase-conjugated anti-mouse secondary antibody (1:5,000; Sigma). Blots were developed using the SuperSignal West Dura extended-duration substrate (Pierce). The signal was detected on X-ray film.

### Overexpression and purification of DgoR-6ХHis

Proteins were expressed and purified, as described previously (26). Briefly, cultures in 400 mL LB were grown at 37°C to an OD_600_ ∼ 0.8, induced with 50 µM IPTG, and incubated again for 12 hours at 18°C. The cells were harvested and resuspended in 20 mL lysis buffer (50 mM Tris [pH 8.5], 1 M NaCl, 1 mM phenylmethylsulfonyl fluoride [PMSF], and 20 mM imidazole). The resuspended cells were sonicated, and cell debris was removed by centrifugation. The supernatant was incubated with Co-NTA beads (Pierce) on ice for 1.5 hours with continuous shaking. Supernatant and beads were then loaded on a column. Beads were successively washed with 30 mL wash buffer A (50 mM Tris [pH 8.5], 1 M NaCl, 10% glycerol, and 20 mM imidazole) and 20 mL wash buffer B (50 mM Tris [pH 8.5], 1 M NaCl, 10% glycerol, and 50 mM imidazole). The protein was eluted in 50 mM Tris (pH 8.5), 1 M NaCl, 10% glycerol, and 500 mM imidazole and dialyzed against 50 mM Tris (pH 8.5), 1 M NaCl, 1 mM dithiothreitol (DTT), and 10% glycerol. To determine protein concentration, absorbance at 280 nm was measured. The concentration of purified DgoR proteins was WT, 50 µM; R71C, 35 µM; and A152E, 8 µM.

### Electrophoretic mobility shift assay

EMSAs were performed as described in reference 26. The 5′-end Cy5-labeled and unlabeled single-stranded complementary oligonucleotides (55 nt), corresponding to the *dgo* promoter, encompassing the DgoR binding site (from Integrated DNA Technologies), were annealed in a ratio of 1:1 to obtain a labeled double-stranded DNA probe. Twenty microliter reactions containing 10 nM labeled double-stranded DNA, 50 mM Tris (pH 8.5), 10 mM MgCl_2_, 265 mM NaCl, 1 mM PMSF, 12% glycerol, 2 mM DTT, and 1 µg herring sperm DNA were set up. Varying concentrations of purified WT or mutant DgoR proteins (0–1.6 µM for WT and R71C; 0–4.2 µM for A152E) were added to the reactions. The reactions were incubated at 27°C for 30 min. For assays involving D-galactonate, DgoR (1 µM for WT and R71C; 3.5 µM for A152E) was incubated with the effector at 37°C for 20 min before incubating with the DNA probe. Ten microliter samples were run on 8% native PAGE gels in 0.5× Tris-borate EDTA buffer, pH 8.3 (45 mM Tris-borate and 1 mM EDTA) at 60 V for 2.5 hours. Fluorescence was detected using ImageQuant LAS4000 (GE Healthcare). Both free and bound DNA were quantified using ImageJ software (57). The apparent *K_D_* value for DgoR DNA interaction was calculated according to the Hill equation using Origin software version 8.5.

### Microscale thermophoresis

MST was performed as described in reference 28. For the *K_D_* measurement of D-galactonate-DgoR interaction, serial dilutions of D-galactonate (100 µM–3.05 nM) and DgoR or its variants (500 nM) were incubated at room temperature for 15 min in a binding buffer (1.8 mM KH_2_PO_4_, 10 mM Na_2_HPO_4_, 137 mM NaCl, 2.7 mM KCl, and 0.05% Tween-20 [pH 7.8]) in a final volume of 20 µL. The samples were then loaded into NT.label-free capillaries (Nano Temper Technologies), and binding experiments were performed on a Monolith NT.label-free instrument (Nano Temper Technologies) with settings: LED power at 20% and MST power set to medium. MST traces were analyzed after activating the IR laser. The MO Control software version 1.6 was used to obtain the results, and the MO Affinity Analysis software version 2.3 was used to determine the fraction of the formed complex. The fraction of the bound complex obtained for each concentration of D-galactonate was plotted against the D-galactonate concentration, and the data were fitted to obtain the apparent *K_D_* (58).

### Generation of structures of wild type and variants

We previously constructed a functional model of the DgoR dimer to gain molecular insights into the influence of D-galactonate binding to the repressor on the release of target DNA (28). The monomeric DgoR exhibits a dual-domain structure with N-terminal DNA-binding and C-terminal E-O domains. To model these domains, we used two separate templates. The DNA-bound structure of the FadR dimer (PDB ID: 1HW2) served as the template for the N-terminal domain and dimeric interface modeling (59). For the C-terminal domain, coordinates from the X-ray structure of the monomeric C-terminal domain of DgoR (PDB ID: 7C7E) were employed (27). Although the PDB ID: 7C7E provides a C-terminal domain orientation via symmetry operation (cyclic-C2, similar to 1HW2), the symmetry-generated dimer of DgoR may not accurately reflect changes due to the missing N-terminal domain and linker regions. Because the linker region and N-terminal domain interactions are critical to our study, the FadR template was used for the N-terminal domain and dimeric interface modeling. PDB ID: 7C7E revealed the presence of a Zn ion within the D-galactonate binding pocket and, thus, was retained in our modeled structure. The generated structure underwent refinement for various loops and minimization using Modeller version 10.4 (60). Here, we introduced single-point mutations, R71C and A152E, in the WT model using Pymol (http://www.pymol.org/pymol). Four sets of simulations were conducted: (i) APO state, (ii) E-bound state, (iii) D-bound state, and (iv) ED-bound state. E-bound and ED-bound initial coordinates were generated by docking minimized structures of APO and D-bound DgoR complexes with D-galactonate using the Autodock Vina module of UCSF Chimera (61). For the DNA coordinates, the FadR DNA sequence was mutated to the inverted repeat sequence of DgoR IR1 (5′-CTAAA**TTGTAGTACAA**CAATAT-3′/5′-ATATTG**TTGTACTACAA**TTTAG-3′) (Fig. S1) (22). The systems underwent a series of minimizations and simulations to facilitate the equilibration of the overall structure.

### Simulation system setup

The exploration involved four distinct simulation sets, as previously described (28). Each system underwent simulation utilizing AMBER forcefields: ff19SB for proteins (62) and OL15 for DNA (63) via GROMACS 2022 (64). A cubic box of TIP3P waters was employed to solvate the systems, neutralized with Na^+^ and Cl^-^ ions, resulting in a simulation size of approximately 80,000 atoms. The solvated neutralized system underwent a process of minimization, equilibration, and a 400 ns simulation period. Long-range interactions were addressed using the Particle Mesh Ewald technique (65), with a non-bonded cutoff set at 12 Å. A 2 fs integration step was applied, and all bonds were constrained using the LINCS algorithm (66, 67). The systems underwent initial minimization using the steepest descent algorithm to eliminate potential clashes and undesirable contacts. This was succeeded by equilibration under the NVT ensemble in two stages. Initially, position restraints of 1,000 kJ/mol/nm^2^ were applied to the heavy atoms of the protein and DNA for 2 ns. During this phase, the minimized structures were gradually heated from 0 to 300 K at a rate of 30 K/100 ps, maintaining the restraints. Subsequently, restraints were exclusively placed on Cα atoms of the protein and backbone atoms of DNA, with a reduced force constant of 100 kJ/mol/nm^2^, for an additional 1 ns of equilibration under the NPT ensemble, holding the simulation temperature at 300 K and pressure at 1 atm. Following the removal of restraints, the final structures underwent further equilibration for 25 ns under the NPT ensemble before entering the production phase. Unrestrained production runs were then conducted for 400 ns at 300 K and 1 atm. Temperature coupling was achieved using the velocity rescaling method with a time constant of 0.1 ps, while pressure coupling utilized the isotropic Parrinello-Rahman pressure coupling algorithm with a time constant of 2.0 and a compressibility value of 4.5e–5 (68). This comprehensive approach resulted in a total of eight simulations for the two variants and an aggregate simulation time of 3.2 μs. For post-trajectory analyses of simulations, please refer to the supplemental material.

### Variation analysis using *E. coli* and Enterobacterales data sets

Homologs of DgoR amino acid sequences were identified using two independent NCBI BLASTp searches against the *E. coli* (taxid: 562) and order *Enterobacterales* (taxid:1347) NR database. The retrieved DgoR protein sequence homologs comprising 229 aa length from each data set were separately aligned and analyzed. In total, 505 *E. coli* DgoR and 1,283 *Enterobacterales* DgoR sequences were included in the analyses. A custom Bash script was developed to identify and quantify variation prevalence within these data sets. For each homologous sequence possessing variation of our interest, respective strain information was further retrieved from the Identical Protein Groups database from NCBI, as shown in Dataset S1.

## Data Availability

The entire data are available in the article and in its online supplemental material.

## SUPPLEMENTAL MATERIAL

Dataset S1 jb.00103-25-s0001.xlsx Prevalence data for P24L, R71C, P92L, and A152E variations.

Supplemental material jb.00103-25-s0002.pdf Supplemental results, methods, figures, tables, dataset, and references.

ASM does not own the copyrights to Supplemental Material that may be linked to, or accessed through, an article. The authors have granted ASM a non-exclusive, world-wide license to publish the Supplemental Material files. Please contact the corresponding author directly for reuse.

## References

[1] BeMiller JN. 2008. Polysaccharides: occurrence, significance, and properties, p 1413–1435. In Fraser-Reid BO, Tatsuta K, Thiem J (ed), Glycoscience: chemistry and chemical biology. Springer, Berlin, Heidelberg.

[2] Brechtel E, Huwig A, Giffhorn F. 2002. L -Glucitol catabolism in Stenotrophomonas maltophilia Ac. 2. Appl Environ Microbiol 68:582–587 68:582–587. doi:10.1128/AEM.68.2.582-587.2002 PMC126675 11823194

[3] Corkins ME, Wilson S, Cocuron JC, Alonso AP, Bird AJ. 2017. The gluconate shunt is an alternative route for directing glucose into the pentose phosphate pathway in fission yeast. J Biol Chem 292:13823–13832. doi:10.1074/jbc.M117.798488 28667014 PMC5566534

[4] Ley JD, Doudoroff M. 1957. The metabolism of D-galactose in Pseudomonas saccharophila. J Biol Chem 227:745–757. doi:10.1016/S0021-9258(18)70755-2 13462997

[5] Ficicioglu C, Hussa C, Gallagher PR, Thomas N, Yager C. 2010. Monitoring of biochemical status in children with Duarte galactosemia: utility of galactose, galactitol, galactonate, and galactose 1-phosphate. Clin Chem 56:1177–1182. doi:10.1373/clinchem.2010.144097 20489133

[6] Lai K, Klapa MI. 2004. Alternative pathways of galactose assimilation: could inverse metabolic engineering provide an alternative to galactosemic patients? Metab Eng 6:239–244. doi:10.1016/j.ymben.2004.01.001 15256214

[7] Stouthamer AH. 1961. Glucose and galactose metabolism in Gluconabacter liquefaciens*.* Biochim Biophys Acta 48:484–500. doi:10.1016/0006-3002(61)90046-4

[8] Conway T, Cohen PS. 2015. Commensal and pathogenic Escherichia coli metabolism in the gut. Microbiol Spectr 3:3. doi:10.1128/microbiolspec.MBP-0006-2014 PMC4510460 26185077

[9] Peekhaus N, Conway T. 1998. What’s for dinner?: entner-doudoroff metabolism in Escherichia coli. J Bacteriol 180:3495–3502. doi:10.1128/JB.180.14.3495-3502.1998 9657988 PMC107313

[10] Sweeney NJ, Laux DC, Cohen PS. 1996. Escherichia coli F-18 and E. coli K-12 eda mutants do not colonize the streptomycin-treated mouse large intestine. Infect Immun 64:3504–3511. doi:10.1128/iai.64.9.3504-3511.1996 8751891 PMC174255

[11] Faber F, Tran L, Byndloss MX, Lopez CA, Velazquez EM, Kerrinnes T, Nuccio SP, Wangdi T, Fiehn O, Tsolis RM, Bäumler AJ. 2016. Host-mediated sugar oxidation promotes post-antibiotic pathogen expansion. Nature 534:697–699. doi:10.1038/nature18597 27309805 PMC4939260

[12] Mandrand-Berthelot MA, Condemine G, Hugouvieux-Cotte-Pattat N. 2004. Catabolism of hexuronides, hexuronates, aldonates, and aldarates. EcoSal Plus 1:10. doi:10.1128/ecosalplus.3.4.2 26443361

[13] Hoskisson PA, Rigali S. 2009. Chapter 1: Variation in form and function, p 1–22. In Laskin AI, Gadd GM, Sariaslani S (ed), Adv Appl Microbiol. Vol. 69. 10.1016/S0065-2164(09)69001-8 19729089

[14] Hoskisson PA, Rigali S, Fowler K, Findlay KC, Buttner MJ. 2006. DevA, a GntR-like transcriptional regulator required for development in Streptomyces coelicolor. J Bacteriol 188:5014–5023. doi:10.1128/JB.00307-06 16816174 PMC1539961

[15] Jain D. 2015. Allosteric control of transcription in GntR family of transcription regulators: a structural overview. 7. IUBMB Life 67:556–563. doi:10.1002/iub.1401 26172911

[16] Rigali S, Derouaux A, Giannotta F, Dusart J. 2002. Subdivision of the helix-turn-helix GntR family of bacterial regulators in the FadR, HutC, MocR, and YtrA subfamilies. J Biol Chem 277:12507–12515. doi:10.1074/jbc.M110968200 11756427

[17] Suvorova IA, Korostelev YD, Gelfand MS. 2015. GntR family of bacterial transcription factors and their DNA binding motifs: structure, positioning and co-evolution. PLoS ONE 10:e0132618. doi:10.1371/journal.pone.0132618 26151451 PMC4494728

[18] Bates Utz C, Nguyen AB, Smalley DJ, Anderson AB, Conway T. 2004. GntP is the Escherichia coli fructuronic acid transporter and belongs to the UxuR regulon. J Bacteriol 186:7690–7696. doi:10.1128/JB.186.22.7690-7696.2004 15516583 PMC524916

[19] Bouvier JT, Sernova NV, Ghasempur S, Rodionova IA, Vetting MW, Al-Obaidi NF, Almo SC, Gerlt JA, Rodionov DA. 2019. Novel metabolic pathways and regulons for hexuronate utilization in Proteobacteria. J Bacteriol 201:e00431-18. doi:10.1128/JB.00431-18 PMC6304669 30249705

[20] Fujita Y, Miwa Y. 1989. Identification of an operator sequence for the Bacillus subtilis gnt operon. J Biol Chem 264:4201–4206. doi:10.1016/S0021-9258(19)84983-9 2492998

[21] Miwa Y, Fujita Y. 1988. Purification and characterization of a repressor for the Bacillus subtilis gnt operon. J Biol Chem 263:13252–13257. doi:10.1016/S0021-9258(18)37698-1 2843515

[22] Singh B, Arya G, Kundu N, Sangwan A, Nongthombam S, Chaba R. 2019. Molecular and functional insights into the regulation of d-galactonate metabolism by the transcriptional regulator DgoR in Escherichia coli. J Bacteriol 201:4. doi:10.1128/JB.00281-18 PMC6351737 30455279

[23] Tutukina MN, Potapova AV, Cole JA, Ozoline ON. 2016. Control of hexuronate metabolism in Escherichia coli by the two interdependent regulators, ExuR and UxuR: derepression by heterodimer formation. Microbiology (Reading, Engl) 162:1220–1231. doi:10.1099/mic.0.000297 27129867

[24] Tutukina MN, Potapova AV, Vlasov PK, Purtov YA, Ozoline ON. 2016. Structural modeling of the ExuR and UxuR transcription factors of E. coli: search for the ligands affecting their regulatory properties. J Biomol Struct Dyn 34:2296–2304. doi:10.1080/07391102.2015.1115779 26549308

[25] Yoshida K, Fujita Y, Sarai A. 1993. Missense mutations in the Bacillus subtilis gnt repressor that diminish operator binding ability. J Mol Biol 231:167–174. doi:10.1006/jmbi.1993.1270 8510140

[26] Arya G, Pal M, Sharma M, Singh B, Singh S, Agrawal V, Chaba R. 2021. Molecular insights into effector binding by DgoR, a GntR/FadR family transcriptional repressor of D-galactonate metabolism in Escherichia coli. Mol Microbiol 115:591–609. doi:10.1111/mmi.14625 33068046

[27] Lin Z, Sun Y, Liu Y, Tong S, Shang Z, Cai Y, Lin W. 2020. Structural and functional analyses of the transcription repressor DgoR from Escherichia coli reveal a divalent metal-containing d-galactonate binding pocket. Front Microbiol 11:590330. doi:10.3389/fmicb.2020.590330 33224125 PMC7674646

[28] Singh S, Arya G, Mishra R, Singla S, Pratap A, Upadhayay K, Sharma M, Chaba R. 2025. Molecular mechanisms underlying allosteric behavior of Escherichia coli DgoR, a GntR/FadR family transcriptional regulator. Nucleic Acids Res 53:gkae1299. doi:10.1093/nar/gkae1299 39777470 PMC11705089

[29] Rosay T, Jimenez AG, Sperandio V. 2024. Glucuronic acid confers colonization advantage to enteric pathogens. Proc Natl Acad Sci USA 121. doi:10.1073/pnas.2400226121 PMC10990124 38502690

[30] Jimenez AG, Ellermann M, Abbott W, Sperandio V. 2020. Diet-derived galacturonic acid regulates virulence and intestinal colonization in enterohaemorrhagic Escherichia coli and Citrobacter rodentium. Nat Microbiol 5:368–378. doi:10.1038/s41564-019-0641-0 31873206 PMC6992478

[31] Eriksson S, Lucchini S, Thompson A, Rhen M, Hinton JCD. 2003. Unravelling the biology of macrophage infection by gene expression profiling of intracellular Salmonella enterica . Mol Microbiol 47:103–118. doi:10.1046/j.1365-2958.2003.03313.x 12492857

[32] Goudeau DM, Parker CT, Zhou Y, Sela S, Kroupitski Y, Brandl MT. 2013. The Salmonella transcriptome in lettuce and cilantro soft rot reveals a niche overlap with the animal host intestine. Appl Environ Microbiol 79:250–262. doi:10.1128/AEM.02290-12 23104408 PMC3536078

[33] Roos V, Ulett GC, Schembri MA, Klemm P. 2006. The asymptomatic bacteriuria Escherichia coli strain 83972 outcompetes uropathogenic E. coli strains in human urine. Infect Immun 74:615–624. doi:10.1128/IAI.74.1.615-624.2006 16369018 PMC1346649

[34] Baron F, Bonnassie S, Alabdeh M, Cochet MF, Nau F, Guérin-Dubiard C, Gautier M, Andrews SC, Jan S. 2017. Global gene-expression analysis of the response of Salmonella Enteritidis to egg white exposure reveals multiple egg white-imposed stress responses. Front Microbiol 8:829. doi:10.3389/fmicb.2017.00829 28553268 PMC5428311

[35] Eberl C, Weiss AS, Jochum LM, Durai Raj AC, Ring D, Hussain S, Herp S, Meng C, Kleigrewe K, Gigl M, Basic M, Stecher B. 2021. E. coli enhance colonization resistance against Salmonella Typhimurium by competing for galactitol, a context-dependent limiting carbon source. Cell Host Microbe 29:1680–1692. doi:10.1016/j.chom.2021.09.004 34610296

[36] Cohen H, Hoede C, Scharte F, Coluzzi C, Cohen E, Shomer I, Mallet L, Holbert S, Serre RF, Schiex T, Virlogeux-Payant I, Grassl GA, Hensel M, Chiapello H, Gal-Mor O. 2022. Intracellular Salmonella Paratyphi A is motile and differs in the expression of flagella-chemotaxis, SPI-1 and carbon utilization pathways in comparison to intracellular S. Typhimurium. PLoS Pathog 18:e1010425. doi:10.1371/journal.ppat.1010425 35381053 PMC9012535

[37] Ku YW, McDonough SP, Palaniappan RUM, Chang CF, Chang YF. 2005. Novel attenuated Salmonella enterica serovar choleraesuis strains as live vaccine candidates generated by signature-tagged mutagenesis. Infect Immun 73:8194–8203. doi:10.1128/IAI.73.12.8194-8203.2005 16299315 PMC1307036

[38] Lescat M, Launay A, Ghalayini M, Magnan M, Glodt J, Pintard C, Dion S, Denamur E, Tenaillon O. 2017. Using long-term experimental evolution to uncover the patterns and determinants of molecular evolution of an Escherichia coli natural isolate in the streptomycin-treated mouse gut. Mol Ecol 26:1802–1817. doi:10.1111/mec.13851 27661780 PMC5734618

[39] Ramiro RS, Durão P, Bank C, Gordo I. 2020. Low mutational load and high mutation rate variation in gut commensal bacteria. PLoS Biol 18:e3000617. doi:10.1371/journal.pbio.3000617 32155146 PMC7064181

[40] Frazão N, Konrad A, Amicone M, Seixas E, Güleresi D, Lässig M, Gordo I. 2022. Two modes of evolution shape bacterial strain diversity in the mammalian gut for thousands of generations. Nat Commun 13:5604. doi:10.1038/s41467-022-33412-8 36153389 PMC9509342

[41] Van Tyne D, Manson AL, Huycke MM, Karanicolas J, Earl AM, Gilmore MS. 2019. Impact of antibiotic treatment and host innate immune pressure on enterococcal adaptation in the human bloodstream. Sci Transl Med 11:487. doi:10.1126/scitranslmed.aat8418 PMC6681809 30971455

[42] Collins J, Robinson C, Danhof H, Knetsch CW, van Leeuwen HC, Lawley TD, Auchtung JM, Britton RA. 2018. Dietary trehalose enhances virulence of epidemic Clostridium difficile. Nature 553:291–294. doi:10.1038/nature25178 29310122 PMC5984069

[43] Minhas V, Harvey RM, McAllister LJ, Seemann T, Syme AE, Baines SL, Paton JC, Trappetti C. 2019. Capacity to utilize raffinose dictates pneumococcal disease phenotype. MBio 10:e02596-18. doi:10.1128/mBio.02596-18 30647157 PMC6336424

[44] Connor CH, Zucoloto AZ, Munnoch JT, Yu IL, Corander J, Hoskisson PA, McDonald B, McNally A. 2023. Multidrug-resistant E. coli encoding high genetic diversity in carbohydrate metabolism genes displace commensal E. coli from the intestinal tract. PLoS Biol 21:e3002329. doi:10.1371/journal.pbio.3002329 37847672 PMC10581457

[45] Singh S, Gola C, Singh B, Agrawal V, Chaba R. 2024. D-galactonate metabolism in enteric bacteria: a molecular and physiological perspective. Curr Opin Microbiol 81:102524. doi:10.1016/j.mib.2024.102524 39137493

[46] Galardini M, Koumoutsi A, Herrera-Dominguez L, Cordero Varela JA, Telzerow A, Wagih O, Wartel M, Clermont O, Denamur E, Typas A, Beltrao P. 2017. Phenotype inference in an Escherichia coli strain panel. Elife 6:e31035. doi:10.7554/eLife.31035 29280730 PMC5745082

[47] Cooper RA. 1978. The utilisation of d-galactonate and d-2-oxo-3-deoxygalactonate by Escherichia coli K-12. Arch Microbiol 118:199–206. doi:10.1007/BF00415730 211976

[48] Monk JM, Charusanti P, Aziz RK, Lerman JA, Premyodhin N, Orth JD, Feist AM, Palsson BØ. 2013. Genome-scale metabolic reconstructions of multiple Escherichia coli strains highlight strain-specific adaptations to nutritional environments . Proc Natl Acad Sci USA 110:20338–20343. doi:10.1073/pnas.1307797110 24277855 PMC3864276

[49] Seif Y, Kavvas E, Lachance JC, Yurkovich JT, Nuccio S-P, Fang X, Catoiu E, Raffatellu M, Palsson BO, Monk JM. 2018. Genome-scale metabolic reconstructions of multiple Salmonella strains reveal serovar-specific metabolic traits. Nat Commun 9:3771. doi:10.1038/s41467-018-06112-5 30218022 PMC6138749

[50] Rancour NJ, Hawkins ED, Wells WW. 1979. Galactose oxidation in liver. Arch Biochem Biophys 193:232–241. doi:10.1016/0003-9861(79)90027-4 453851

[51] Berry GT, Wehrli S, Reynolds R, Palmieri M, Frangos M, Williamson JR, Segal S. 1998. Elevation of erythrocyte redox potential linked to galactonate biosynthesis: elimination by Tolrestat. Metab Clin Exp 47:1423–1428. doi:10.1016/S0026-0495(98)90317-1 9826225

[52] Yager C, Ning C, Reynolds R, Leslie N, Segal S. 2004. Galactitol and galactonate accumulation in heart and skeletal muscle of mice with deficiency of galactose-1-phosphate uridyltransferase. Mol Genet Metab 81:105–111. doi:10.1016/j.ymgme.2003.10.001 14741191

[53] Saffarian A, Mulet C, Naito T, Bouchier C, Tichit M, Ma L, Grompone G, Sansonetti PJ, Pédron T. 2015. Draft genome sequences of acinetobacter parvus cm11, acinetobacter radioresistens cm38, and stenotrophomonas maltophilia br12, isolated from murine proximal colonic tissue. Genome Announc 3:e01089-15. doi:10.1128/genomeA.01089-15 26472823 PMC4611675

[54] Wale KR, O’Boyle N, McHugh RE, Serrano E, Mark DR, Douce GR, Connolly JPR, Roe AJ. 2024. A master regulator of central carbon metabolism directly activates virulence gene expression in attaching and effacing pathogens. PLoS Pathog 20:e1012451. doi:10.1371/journal.ppat.1012451 39405360 PMC11508082

[55] Pifer R, Russell RM, Kumar A, Curtis MM, Sperandio V. 2018. Redox, amino acid, and fatty acid metabolism intersect with bacterial virulence in the gut. Proc Natl Acad Sci USA 115. doi:10.1073/pnas.1813451115 PMC6233112 30348782

[56] Egan M, Ramirez J, Xander C, Upreti C, Bhatt S. 2016. Lambda red-mediated recombineering in the attaching and effacing pathogen Escherichia albertii. Biol Proced Online 18:1. doi:10.1186/s12575-015-0032-8 26843851 PMC4739404

[57] Schneider CA, Rasband WS, Eliceiri KW. 2012. NIH Image to ImageJ: 25 years of image analysis. Nat Methods 9:671–675. doi:10.1038/nmeth.2089 22930834 PMC5554542

[58] Seidel SAI, Wienken CJ, Geissler S, Jerabek-Willemsen M, Duhr S, Reiter A, Trauner D, Braun D, Baaske P. 2012. Label-free microscale thermophoresis discriminates sites and affinity of protein-ligand binding. Angew Chem Int Ed Engl 51:10656–10659. doi:10.1002/anie.201204268 23001866 PMC3588113

[59] Xu Y, Heath RJ, Li Z, Rock CO, White SW. 2001. The FadR·DNA Complex. Journal of Biological Chemistry 276:17373–17379. doi:10.1074/jbc.M100195200 11279025

[60] Webb B, Sali A. 2016. Comparative protein structure modeling using modeller. Curr Protoc Bioinformatics 54:5. doi:10.1002/cpbi.3 PMC5031415 27322406

[61] Pettersen EF, Goddard TD, Huang CC, Couch GS, Greenblatt DM, Meng EC, Ferrin TE. 2004. UCSF Chimera--a visualization system for exploratory research and analysis. J Comput Chem 25:1605–1612. doi:10.1002/jcc.20084 15264254

[62] Tian C, Kasavajhala K, Belfon KAA, Raguette L, Huang H, Migues AN, Bickel J, Wang Y, Pincay J, Wu Q, Simmerling C. 2020. ff19SB: Amino-acid-specific protein backbone parameters trained against quantum mechanics energy surfaces in solution. J Chem Theory Comput 16:528–552. doi:10.1021/acs.jctc.9b00591 31714766 PMC13071887

[63] Galindo-Murillo R, Robertson JC, Zgarbová M, Šponer J, Otyepka M, Jurečka P, Cheatham TE. 2016. Assessing the current state of amber force field modifications for DNA. J Chem Theory Comput 12:4114–4127. doi:10.1021/acs.jctc.6b00186 27300587 PMC4980684

[64] Abraham MJ, Murtola T, Schulz R, Páll S, Smith JC, Hess B, Lindahl E. 2015. GROMACS: High performance molecular simulations through multi-level parallelism from laptops to supercomputers. SoftwareX 1–2:19–25. doi:10.1016/j.softx.2015.06.001

[65] Darden T, York D, Pedersen L. 1993. Particle mesh Ewald: An N ⋅log(N) method for Ewald sums in large systems. 12. J Chem Phys 98:10089–10092. doi:10.1063/1.464397

[66] Hess B. 2008. P-LINCS: a parallel linear constraint solver for molecular simulation. J Chem Theory Comput 4:116–122. doi:10.1021/ct700200b 26619985

[67] Hess B, Bekker H, Berendsen HJC, Fraaije JGEM. 1997. LINCS: A linear constraint solver for molecular simulations. J Comput Chem 18:1463–1472. doi:10.1002/(SICI)1096-987X(199709)18:12<1463::AID-JCC4>3.0.CO;2-H

[68] Bussi G, Donadio D, Parrinello M. 2007. Canonical sampling through velocity rescaling. J Chem Phys 126:014101. doi:10.1063/1.2408420 17212484

