# Supplementary material for: Functional consequences of genetic variations in DgoR, a GntR/FadR family transcriptional repressor of D-galactonate metabolism in Escherichia coli
Source: J Bacteriol. 2025 Jun 11;207(7):e00103-25. doi: 10.1128/jb.00103-25 (PMC12288454; doi:10.1128/jb.00103-25)
Supplement: Supplemental material — Supplemental results, methods, figures, tables, dataset, and references. [file jb.00103-25-s0002.pdf]

Supplemental Material

Functional consequences of genetic variations in DgoR, a GntR/FadR family transcriptional repressor of D-galactonate metabolism in *Escherichia coli*

Swati Singh[1], Rajesh Mishra[1], Richa Ashok Kakkar[2], Shivam Singla[1], Akhil Pratap[3], Gaurav Sharma[2], Monika Sharma[3], and Rachna Chaba[1,*]

[1] Department of Biological Sciences, Indian Institute of Science Education and Research (IISER) Mohali, SAS Nagar, Punjab, 140306, India

[2] Department of Biotechnology, Indian Institute of Technology Hyderabad, Sangareddy, Telangana, 502285, India

[3] Biological Systems Engineering, Plaksha University, SAS Nagar, Punjab, 140306, India

* To whom correspondence should be addressed. Tel: (91)-8146084997; Fax: (91)-1722240266; Email: rachnachaba@iisermohali.ac.in; rachnachaba@gmail.com

SUPPLEMENTAL RESULTS

Detailed analyses of molecular dynamics (MD) simulations

Variations alter the deviations and correlations of DgoR complexes

We assessed the comparative structure stabilities of different simulated states of wild-type (WT) DgoR using root mean square deviation (RMSD) and root mean square fluctuation (RMSF) values. We observed higher RMSD values for dimeric full-length protein in APO and ED-bound states (Fig. S6A). For the N-terminal domain, the RMSF values are higher in APO and ED-bound states. For the C-terminal domain, RMSF values are higher in E-bound and ED-bound states, indicating conformational changes upon effector binding (Fig. S6B). RMSD values for both variants are higher in the E-bound state than their other states, primarily for A152E (Fig. S6A), indicating that the variations affect the overall conformational dynamics of the DgoR complex in the presence of an effector. RMSF values of both variants are higher for APO and E-bound states compared to their D-bound and ED-bound states (Fig. S6B).

The transmission of an allosteric signal upon DNA binding to the N-terminal domain or effector binding to the C-terminal domain should couple motions within various domains of DgoR. We, therefore, computed the dynamic cross-correlation (DCC) coefficients for the Cα atom of each residue and generated DCC maps for WT and variants (Fig. S7). For WT, we observed anti-correlations between the N-terminal (Region A) and C-terminal domains (Region B) of two monomers in the APO state. In the E-bound state, both positive and negative correlations increased for Regions A and B, suggesting an allosteric effect of effector binding in the C-terminal domain to the movement of the N-terminal domain of DgoR. In the D-bound state, positive correlations were seen in Region A, and a decrease in anti-correlations was observed

in Region B. In the ED-bound state, anti-correlations intensified, resembling the APO state. These findings suggest that effector binding in the C-terminal domain allosterically modulates the N-terminal domain, facilitating DNA release. The comparative DCC maps of the DgoR variants showed differences in correlation patterns attributable to the variations, suggesting the effect of variations on allosteric communication. For R71C, comparing APO and E-bound states, similar to the WT, anti-correlations intensified in the E-bound state; however, the extent of anti-correlations overall is more in E-bound R71C than in E-bound WT. The sparsely distributed correlation islands in the D-bound state of R71C indicate structural stabilization when DNA is bound to the N-terminal domain. Comparing D-bound and ED-bound states, although anti-correlations increase in ED-bound R71C, the extent of the increase is less than ED-bound WT, indicating rigidity that likely hinders DNA release, thereby underscoring the impact of the R71C variation on effector interaction dynamics. For A152E, when comparing Regions C-F of the D-bound state with the D-bound state of WT, distinct correlation patterns are observed for Regions C and D and Regions E and F. Thus, DNA binding to A152E does not induce effective synchronized motions, hence A152E does not effectively interact with DNA. However, the correlations in E-bound and ED-bound states are similar to WT, suggesting similar behavior for this variant as WT.

Variations affect the essential dynamics and networks of DgoR complexes

MD simulations revealed long-range correlated motions in the topmost principal component (PC) (Fig. 6A). For WT DgoR, in the APO state, significant movements were observed in the N-terminal domains. In the E-bound state, synchronized movements between the N-terminal and C-terminal domains indicated allosteric

interactions. In the D-bound state, there were no significant motions. However, in the ED-bound state, significant rotational motions occur in the N-terminal domain, particularly in the wHTH motif, suggesting allosteric regulation of DNA binding. We compared the motion along PC1 in the variants across different states. In the APO state, R71C exhibited less significant motions than A152E and WT, while A152E showed notable motions, indicating flexibility in the APO state. In the E-bound and D-bound states, similar motions were observed for both WT and the variants, with significant motion in the N-terminal domain of R71C in the E-bound state. In the ED-bound state, distinct patterns emerged, with WT showing the most pronounced motion, followed by moderate motion in A152E and no significant motion in R71C. These observations suggest that whereas effector binding would facilitate DNA release by WT and A152E, restricted motion in R71C may disfavor DNA release.

Variations affect the dynamic network involved in allosteric communication

Next, we turned to the dynamical network analysis to observe communication pathways. For this, we integrated the correlation data obtained from MD simulations with structure-based network analysis (Fig. 6B). In the APO state of WT DgoR, 10 distinct communities were identified, with residues from different domains forming separate communities. In the E-bound state, an additional community formed in the N-terminal region, resulting in 11 communities. In the D-bound state, 10 communities persisted, but with a notable rearrangement of residues, especially in the N-terminal domain, which incorporated nucleotides from the bound DNA to form new communities. In the ED-bound state, the number of communities increased to 13, with the DNA nucleotides forming distinct communities separate from the N-terminal residues. For R71C, we observed 12 distinct communities in the APO state that

reduced to 10 in the E-bound state and increased to 14 in the D-bound state. For A152E, we observed 9 communities for APO, E-bound, and D-bound states, and though the number remains the same, rearrangements are observed to accommodate the bound effector and DNA. ED-bound states of R71C and A152E formed 12 and 10 communities, respectively. For R71C, DNA nucleotides form a community with the N-terminal domain in the ED-bound state, suggesting that this variant will affect DNA release. However, no community within the DNA and N-terminal domain is observed in the ED-bound state of A152E, suggesting the DNA-releasing capability of this variant.

To examine the effect of variations on allosteric communication, the total number of suboptimal paths and distributions of suboptimal path lengths were computed for various states of WT and DgoR variants (Fig. 6C and D). The number of suboptimal paths suggests the number of ways through which the signal or information can pass. An increase in the number of suboptimal paths can increase the efficiency of communication by increasing the number of available communication pathways or lead to reduced efficiency if the signal or information has to pass through more intermediates. Thus, we also looked at the distributions of suboptimal path lengths. A right shift in the plot suggests longer suboptimal path lengths and, thus, a delay in passing the information, while a left shift suggests shorter suboptimal path lengths, suggesting a quicker communication (Fig. 6D).

We observed that in all four states of WT DgoR, allosteric communication involved residues from both monomers. The highest number of suboptimal paths, 1032, was in the APO state, which decreased to 405 in the E-bound state, with fewer interconnecting nodes needed for communication. The number of paths reduced to 523 in the D-bound state. In the ED-bound state, the number of paths increased to

681, indicating that effector binding induces alternate allosteric pathways, facilitating DNA release (Fig. 6C). Despite variations in the number of paths, the path length distribution remained consistent across all states, ensuring efficient allosteric communication in WT DgoR (Fig. 6D). Compared to the WT, the total number of suboptimal paths decreased and increased in the APO states of R71C and A152E, respectively. The number of suboptimal paths increased in the E-bound states of both R71C and A152E, with a significantly higher number for A152E. In the D-bound state, the number of suboptimal paths reduced in R71C, while it remained similar in A152E. Interestingly, the number of suboptimal paths decreased in the ED-bound states of both R71C and A152E (Fig. 6C). We observed that for the R71C variant, there is a right shift for the E-bound state and a left shift for the D-bound state when compared to the E-bound and D-bound states of WT (Fig. 6D). Combining the right shift in the E-bound state with the increase in the number of suboptimal paths suggests that the R71C variant may affect the effector-binding ability of DgoR. On the other hand, combining the left shift in the D-bound state with the decrease in the number of suboptimal paths suggests that the R71C variant may not affect the DNA-binding ability. Compared to WT, we observed the right shift for APO, E-bound, and D-bound states of A152E. Mapping this with the number of suboptimal paths for A152E, we observe that this variant effectively communicates when bound to the effector compared to when bound to DNA. Thus, A152E will favor effector binding over DNA binding. We observed a left shift for the ED-bound state of A152E in the distribution of suboptimal path lengths, which indicates that despite the reduction in the number of suboptimal paths, the effector binding to the C-terminal domain will still regulate the DNA release ability of the N-terminal domain (Fig. 6C and D).

SUPPLEMENTAL MATERIALS AND METHODS

Circular dichroism (CD) measurements

Far-UV CD spectra of WT and variant DgoR-6XHis proteins (WT and R71C- 5 μM, and A152E- 2.5 μM) [in buffer containing 20 mM Tris (pH 8.5), 300 mM NaCl, 1 mM dithiothreitol (DTT), and 10% glycerol] were recorded (1). The spectra were collected on a Chirascan spectrophotometer (Applied Photophysics, UK) at 25°C. The scans were collected from 200-260 nm with a step size of 1 nm using a quartz cuvette with a path length of 1 mm. In each experiment, the spectra were recorded in triplicate, and their average values were blank subtracted.

Analytical size exclusion chromatography

The WT and variant DgoR-6XHis proteins [WT- 15 μM and R71C- 10 μM in 50 mM Tris (pH 8.5), 300 mM NaCl, 1 mM DTT, and 10% glycerol, and A152E- 8 μM in 50 mM Tris (pH 8.5), 1M NaCl, 1 mM DTT, and 10% glycerol] were loaded on a Superdex 75 Increase 10/300 GL column (GE Healthcare, Munich, Germany) equilibrated with a mobile phase [50 mM Tris (pH 8.5), 300 mM NaCl, 1 mM DTT, and 10% glycerol]. The runs were performed at a flow rate of 0.3 ml min^{-1} (1). The calibration curve for the column is reported in (2).

Structure deviation and correlation analyses

Structural analyses, encompassing RMSD and RMSF, were conducted employing VMD-1.9.4 (http://www.ks.uiuc.edu/Research/vmd/) (3). RMSD calculations involved all backbone atoms, with the experimental structure serving as the reference. For RMSF, Cα atoms in all trajectories were considered. These calculations were executed using a custom tcl script for VMD.

To gauge the extent of correlated motion between two residues, the magnitude of the cross-correlation C_{ij} for each pair of Cα atoms of residues i and j was computed as follows: $C_{ij} = \frac{\langle \Delta r_i . \Delta r_j \rangle}{\langle \Delta r_i^2 \rangle^{1/2} . \langle \Delta r_j^2 \rangle^{1/2}}$, where r_{ij} is the displacement from the mean position of atom i, and symbol <> denotes the time-average. DCC analyses were performed using the Bio3D package (4) for Cα atoms, pooling all conformations sampled at 2 ps intervals across multiple simulation runs.

Principal component (PC) and network analyses

PC analyses were conducted using the Bio3D package implemented in R. A variance-covariance matrix was generated by aggregating Cα atoms from all conformations sampled at 5 ps intervals across the trajectories. Time-dependent projections of the first principal components onto the trajectories were then examined.

The identification of allosteric networks within the proteins utilized the NetworkView plugin of VMD-1.94 (5). Dynamical networks were constructed using data from all trajectories of the protein-DNA complexes, sampled at 5 ps intervals. For each molecular system, a network graph was created with two nodes for each nucleotide molecule. N1 and N9 atoms defined the nodes for the base atoms of pyrimidines and purines, respectively, while the phosphorus (P) atom was used for the nodes representing sugar and backbone phosphate atoms. Protein residue nodes were defined by Cα atoms. Conformations were pooled to calculate the local contact matrix, establishing a contact between two nodes (excluding neighboring nodes) within 4.5 Å, observed for at least 75% of the simulation time. The constructed contact matrix was weighted by the correlation values of the two end nodes, denoted as C_{ij}, in the correlation matrix. The community structure was identified using the Girvan-Newman

algorithm, employing edge betweenness to detect community peripheries (6). Edge betweenness measures the number of shortest paths between pairs of nodes running along a specific edge, indicating the influence of a node over the flow of information between other nodes. The iterative process of community detection involved calculating the betweenness of all edges in the network, followed by the removal of the edge with the highest betweenness until no edges remained.

To investigate allostery within the network, suboptimal path analyses were performed between the N-terminal and C-terminal domains. For the N-terminal domain, we selected residues interacting with DNA: A32, S41, M60, Y63, and A66, and for the C-terminal domain, we selected residues lining the effector binding cavity: E106, F142, H150, F177, H195, and S221. An edge length offset of 5 was used to calculate the suboptimal paths between these residues.

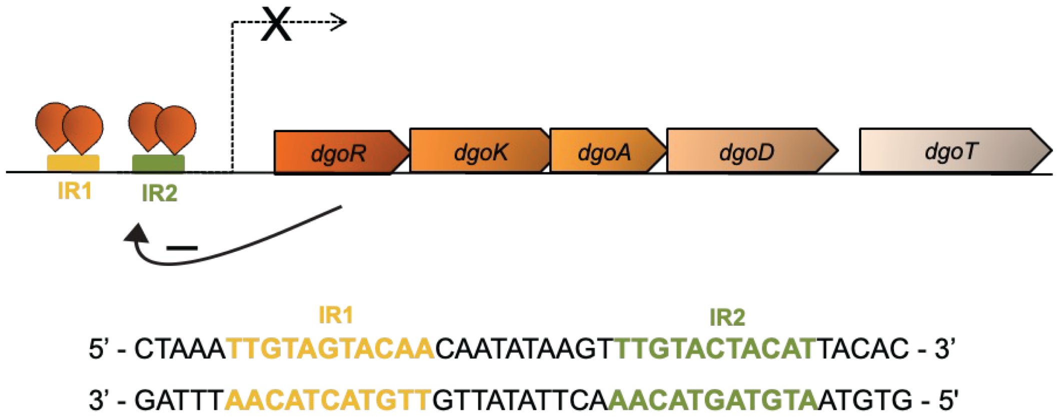

Figure S1. *Model depicting the repression of dgo operon by DgoR.* The transcriptional repressor, DgoR, is encoded by the first gene of the *dgo* operon, which also harbors structural genes involved in D-galactonate transport (*dgoT*) and metabolism (*dgoK*, *dgoA*, and *dgoD*). DgoR represses the *dgo* operon by binding to two closely spaced inverted repeats (IR1 and IR2) in the *cis*-acting element that overlap with the *dgo* promoter. GntR family proteins usually bind to the inverted repeat as dimers, where each monomer recognizes a half-site. As DgoR also forms dimers, two DgoR homodimers likely bind the *dgo* promoter. Solid arrows (not drawn to scale) indicate the direction of *dgo* genes and the bent dashed arrow denotes the direction of transcription. Lower panel: The *dgo cis*-acting element sequence that encompasses the two inverted repeats is shown. IR1 is a perfect inverted repeat, whereas IR2 has one mismatch.

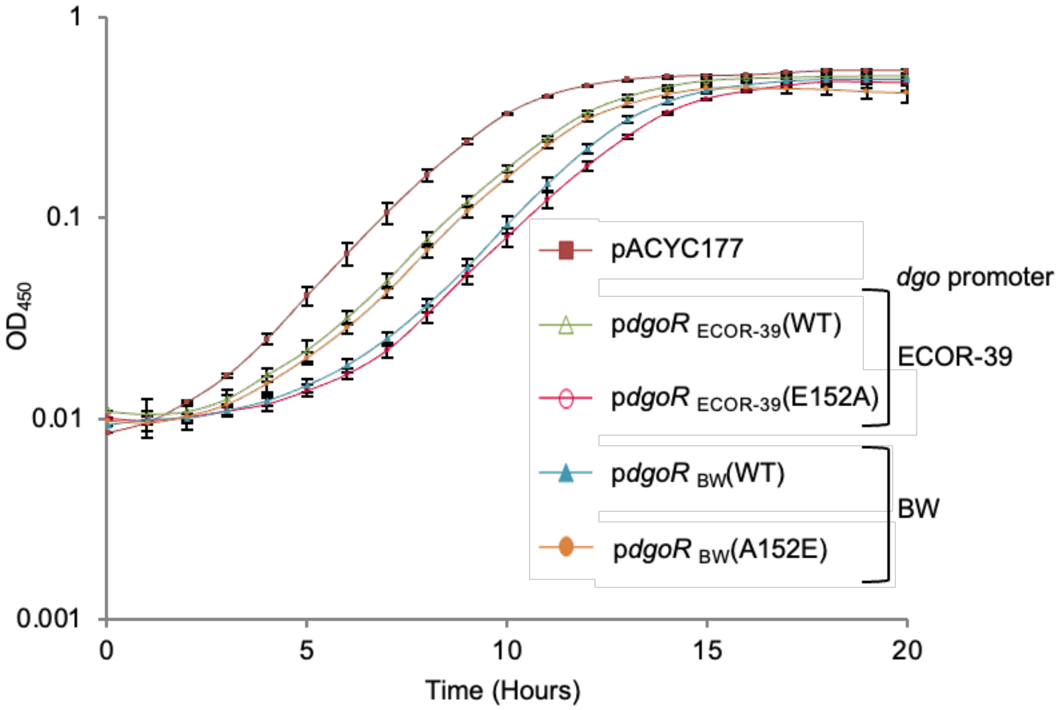

Figure S2. *A152E variation in DgoR results in the faster growth of ECOR-39 in D-galactonate.* The plasmid pACYC177, pACYC177 carrying BW *dgoR* cloned under BW *dgo* promoter [p*dgoR*$_{BW}$(WT), pBS13 and p*dgoR*$_{BW}$(A152E), pSW26] and pACYC177 carrying ECOR-39 *dgoR* cloned under ECOR-39 *dgo* promoter [p*dgoR*$_{ECOR\text{-}39}$(WT), pSW24 and p*dgoR*$_{ECOR\text{-}39}$(E152A), pSW25] were individually transformed in two parental backgrounds of the ECOR-39 *dgoR*::*kan* strain (Parents 1 and 2). Cultures were grown in an M9 minimal medium supplemented with D-galactonate as the sole carbon source, and OD_{450} was measured. The experiment was performed two times, each with transformants of Parent 1 (Fig. 4A) and Parent 2; each experiment had three technical replicates. A representative data set, with average (±SD) from technical replicates, for transformants of Parent 2 is shown. Note: Because P1 transduction was not successful in the ECOR-39 strain, we performed the above experiment in two parental backgrounds to validate the phenotypes.

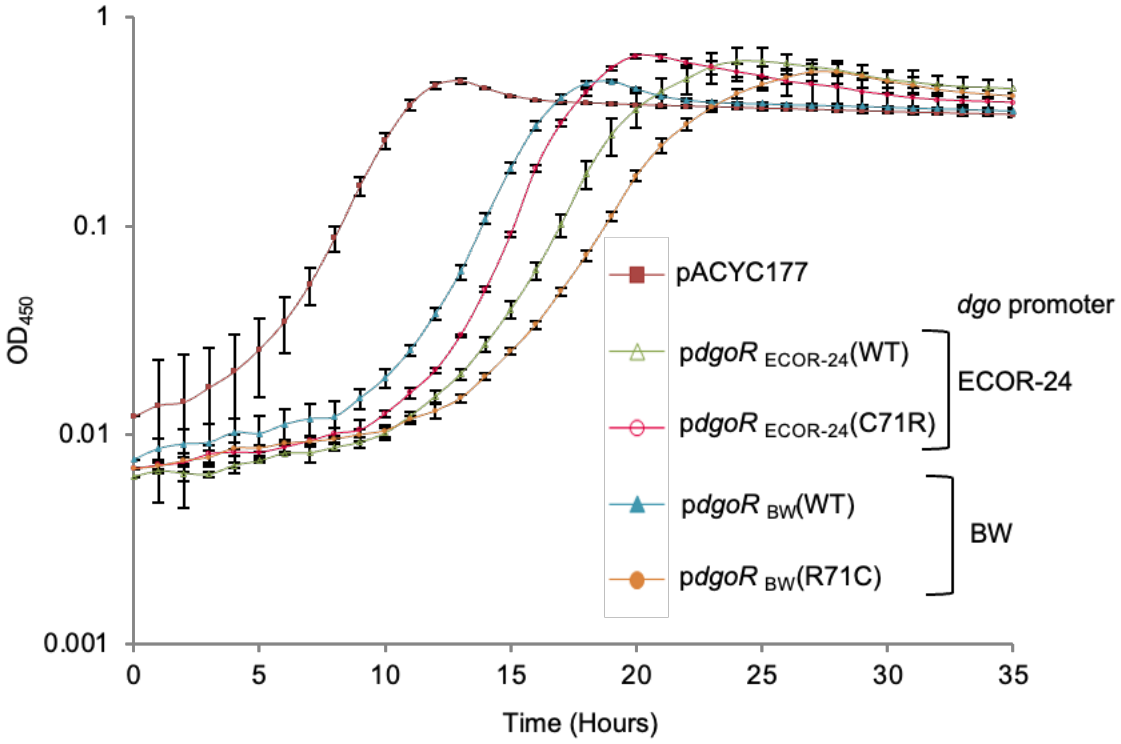

Figure S3. *R71C variation in DgoR results in the slower growth of ECOR-24 in D-galactonate.* The empty plasmid pACYC177, pACYC177 carrying BW *dgoR* cloned under BW *dgo* promoter [p*dgoR*$_{BW}$(WT), pBS13 and p*dgoR*$_{BW}$(R71C), pSW28] and pACYC177 carrying ECOR-24 *dgoR* cloned under ECOR-24 *dgo* promoter [p*dgoR*$_{ECOR-24}$(WT), pSW35 and p*dgoR*$_{ECOR-24}$(C71R), pSW38] were individually transformed in two parental backgrounds of the ECOR-24 *dgoR*::*kan* strain (Parents 1 and 2). Cultures were grown in an M9 minimal medium supplemented with D-galactonate as the sole carbon source, and OD_{450} was measured. The experiment was performed two times, each with transformants of Parent 1 (Fig. 4B) and Parent 2; each experiment had three technical replicates. A representative data set, with average (±SD) from technical replicates, for transformants of Parent 2 is shown. Note: Because P1 transduction was not successful in the ECOR-24 strain, we performed the above experiment in two parental backgrounds to validate the phenotypes.

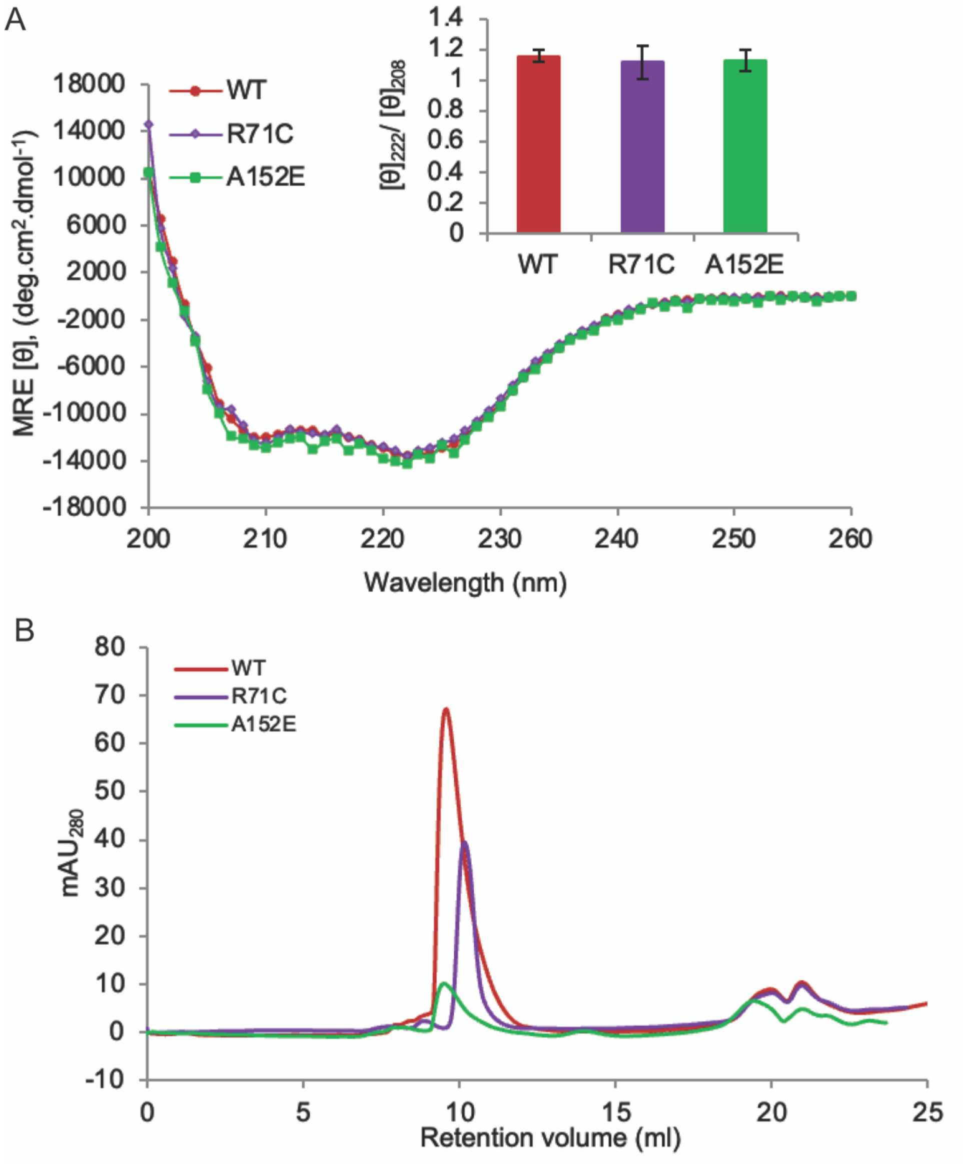

Figure S4. (A) R71C and A152E variants are folded. Far-UV CD spectra of BW WT DgoR-6XHis and its variants were recorded. The experiment was performed three times. A representative data set is shown. *Inset:* Mean residue ellipticity ratios $[\theta]_{222}/[\theta]_{208}$ for WT DgoR and variants. $[\theta]_{222}/[\theta]_{208}$ values are the average (±SD) of three independent experiments and are similar for all the proteins. (B) R71C and A152E variants have a similar retention volume on a size exclusion column as the BW WT protein. WT DgoR-6XHis and its variants were loaded on a Superdex 75 Increase column, and their elution profile was recorded by measuring absorbance at 280 nm. The proteins eluted as a single major peak with a similar retention volume. The

experiment was performed two times. A representative data set is shown. The concentration of the purified proteins loaded on the column was different (see Supplemental Materials and Methods).

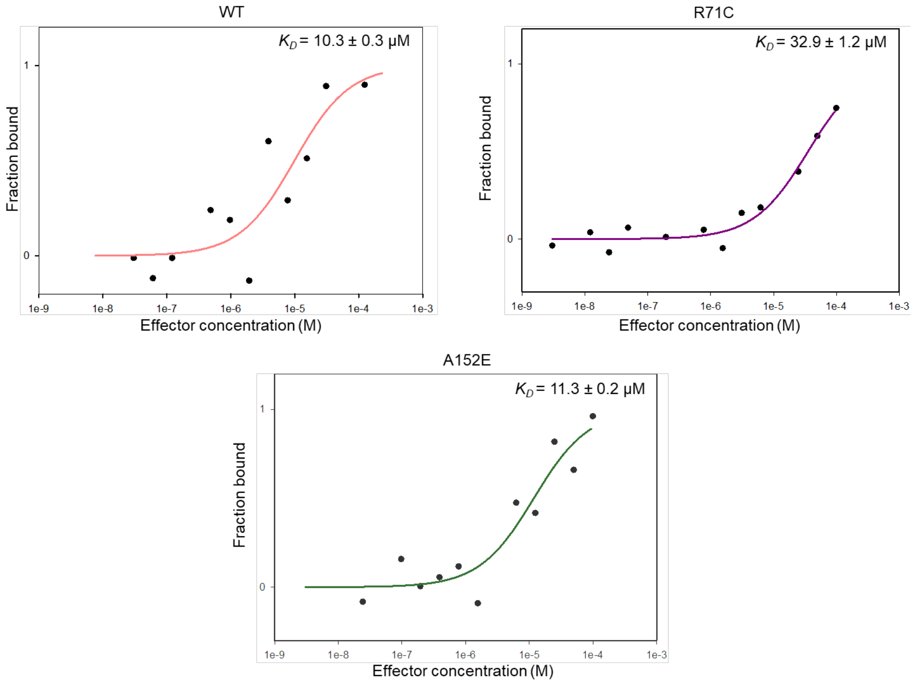

Figure S5. *R71C variant has a weaker affinity for D-galactonate.* Related to Fig. 5D. Serially diluted D-galactonate (100 μM – 3.05 nM) and BW WT DgoR-6XHis or its variants (500 nM) were incubated for 15 min at room temperature. The samples were then loaded into label-free capillaries. Binding assays were conducted, and data were analyzed and plotted as described in Materials and Methods. The experiments were performed three times for each protein. A representative MST plot is shown. The average K_D values (±SD) from three independent experiments for each protein are shown along with their representative MST plot.

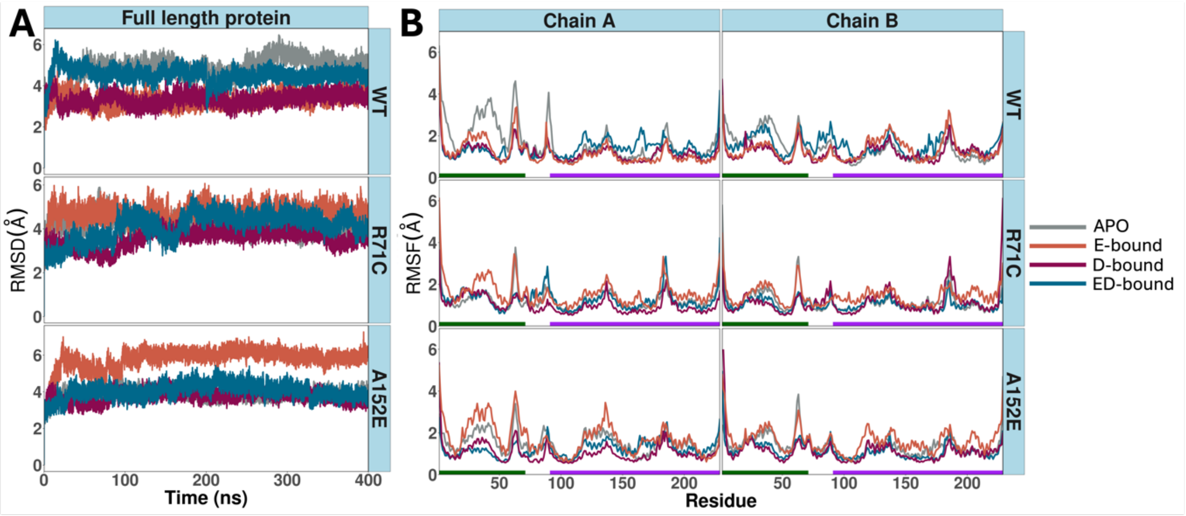

Figure S6. (A) Time-evolution plots of RMSD values of backbone atoms of dimeric full-length DgoR calculated for different states of WT and variants. (B) RMSF values of different states of DgoR dimeric complexes. Residues 1-71 and 91-229 are annotated as N-terminal and C-terminal domains, respectively, and residues 72-90 are annotated as the linker region. The N-terminal and C-terminal domain residues are annotated with green and purple-colored boxes along the X-axis, respectively.

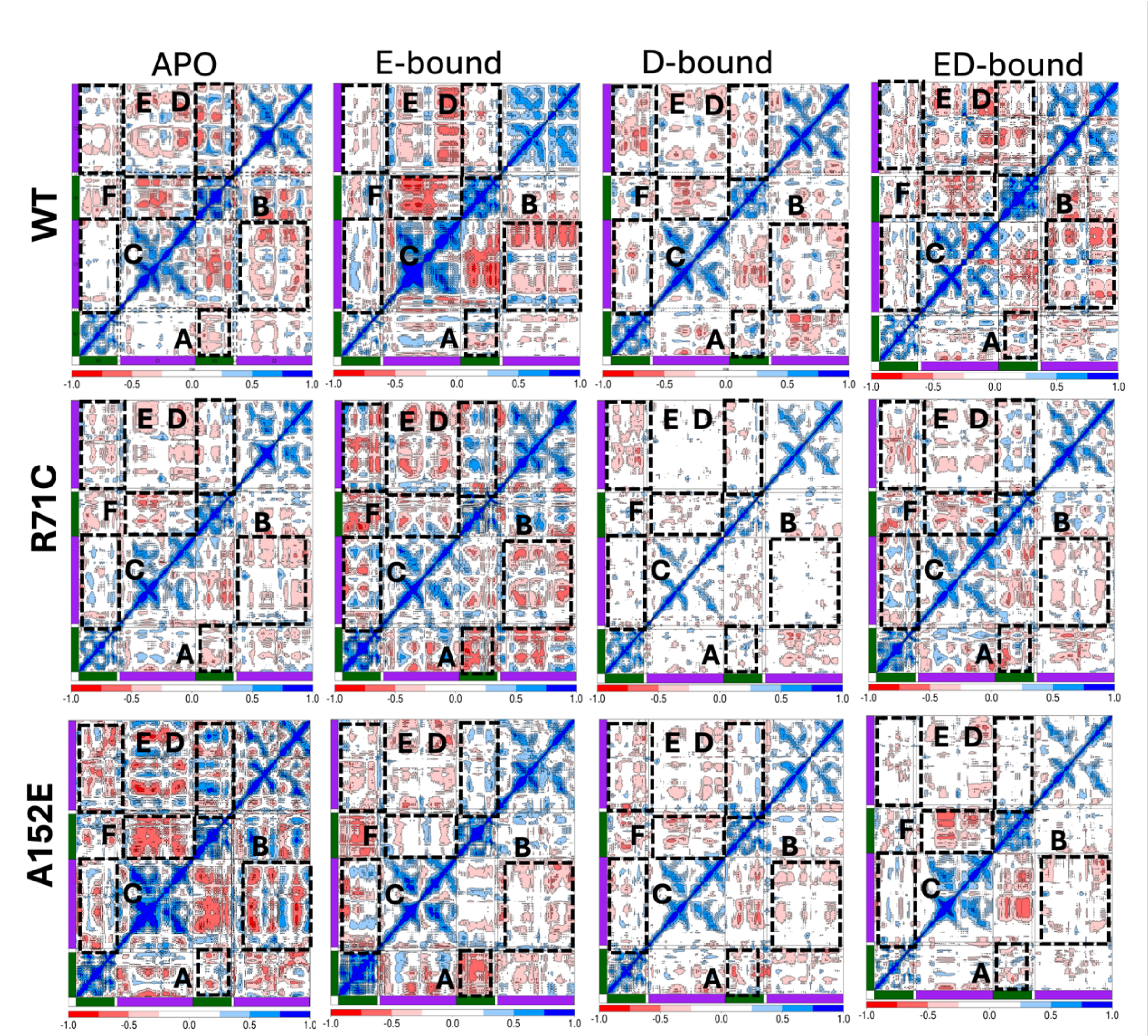

Figure S7. DCC maps calculated as the time-average for Cα atoms of dimeric protein for WT and variant DgoR complexes. The N-terminal (residues 1-71) and C-terminal (residues 91-229) domains are annotated with green- and purple-colored boxes, respectively. Correlation coefficients are color-coded; the color bar is indicated at the end of each map. Correlation coefficients range from -1 to +1; blue for positive correlations (0.25 to 1), red for negative correlations (-0.25 to -1), and white for weak or no correlations (-0.25 to +0.25). Regions (A-F), distinctly marked on all DCC maps, show correlations between different structural regions: (i) Region A: inter-correlations between the N-terminal domains of the DgoR dimer; (ii) Region B: inter-correlations between the C-terminal domains of the DgoR dimer; (iii) Regions C and D: intra-

correlations between the N-terminal and C-terminal domains of the same monomer; and (iv) Regions E and F: inter-correlations between a domain of one monomer and another domain of second monomer.

SUPPLEMENTAL TABLES

Table S1 List of natural *E. coli* isolates with amino acid variations in DgoR (7)

**Amino acid variation in DgoR (in comparison to *E. coli* BW25113)**	**Strain name (*E. coli* natural isolate)**	**Phenotype**	**Isolation origin**
L3F	ECOR-53	Commensal	Faeces of healthy female (child), USA (Iowa)
	IAI77	Pathogenic	Human urine
	NILS 70	Pathogenic	Human urine
K15R	ED1a	Commensal	Human faeces
	MP1	Commensal	Mouse faeces
	ZG-46.1	Commensal	Healthy human adults, Zielona Góra, Poland
	ZG-46.5	Commensal	Healthy human adults, Zielona Góra, Poland
	ZG-50.1	Commensal	Healthy human adults, Zielona Góra, Poland
	DE-COMM-2574	Commensal	Squirrel
	DE-COMM-2584*	Commensal	Squirrel
	DE-COMM-2703	Commensal	Roe deer
	DE-COMM-4123	Commensal	Brown rat
	H001	Commensal	Human, blood stream, Canberra, ACT, Australia
	TA103	Commensal	Mitchell's hopping mouse, Wyperfeld National Park, VIC, Australia
	IAI48	Pathogenic	Human blood
	IAI55	Pathogenic	Human urine
	IAI70	Pathogenic	Human urine
	NILS 20	Pathogenic	Human blood
	NILS 81	Pathogenic	Human urine
	NR-15878	Unknown	-
	ECOR-65x	-	-
H19Q	DE-COMM-2584*	Commensal	Squirrel
P24L	HM-50	Commensal	Asymptomatic human urine, Sweden
	UTI 83972	Commensal	Asymptomatic human urine, Sweden
**R71C**	**ECOR-24**	**Commensal**	**Faeces of healthy women, Sweden**
	IAI13	Commensal	Human faeces
P92L	NILS 56[#]	Pathogenic	Human urine
A128S	ECOR-50	Commensal	Urine of women with UTI, Sweden
	NILS 56[#]	Pathogenic	Human urine

	ECOR-49x	-	-
**A152E**	**ECOR-39**	**Commensal**	**Faeces of healthy women, Sweden**
	ECOR-40	Commensal	Urine of women with UTI, Sweden
T180S	DE-COMM-4965	Commensal	Human
	DE-COMM-4973	Commensal	Human
	HM-344	Pathogenic	Patient with ulcerative colitis, New York, USA
	ECOR-22x	-	-
	ECOR-23x	-	-
A186V	DE-COMM-4979	Commensal	Human
	ZG-44.1	Commensal	Healthy human adults, Zielona Góra, Poland
	NILS 63	Pathogenic	Human urine
	IAI64x	-	-
S220C	O08	Pathogenic	Yolk of the abdomen of a diseased 1-day-old chick, Brasil
T222I	HM-346	Pathogenic	Patient with Crohn's disease, New York, USA
	IAI35	Pathogenic	Human blood

* DE-COMM-2584 strain has K15R and H19Q variations in DgoR.

# NILS 56 strain has P92L and A128S variations in DgoR.

Strains highlighted in bold have been used in this study.

Table S2 Strains and plasmids used in this study

Strains/plasmids	*Relevant genotype/ Relevant characteristics/ Description*	*Source/reference*
Strains		
DH5α	F$^-$ Δ(*argF-lac*)*169* ϕ80d*lacZ58*(M15) *glnX44*(AS) λ$^-$ *rfbC1* *gyrA96*(Nal^r) *recA1* *endA1* *spoT1* *thiE1* *hsdR17*	New England Biolabs
BL21(DE3)	F$^-$ *lon-11* Δ(*ompT-nfrA*)*885* Δ(*galM-ybhJ*)*884* λDE3 [*lacI lacUV5-T7* gene 1 *ind1 sam7 nin5*] Δ*46* [*mal*$^+$]$_{K-12}$(λ^S) *hsdS10*	New England Biolabs
BW25113	F$^-$ Δ(*araD-araB*)*567* Δ*lacZ4787*(::*rrnB-3*) λ$^-$ *rph-1* Δ(*rhaD-rhaB*)*568* *hsdR514*	CGSC[‡] (8)
dgoR::*kan*	BW25113 *dgoR*::*kan* Kan^r	Keio collection (8)
RC2145	Δ*dgoA* (Kan *cassette* flipped from BW25113 *dgoA*::*kan*)	(1)
RC12022	BW25113 *attλ*::[Kan promoterless-*venus oriR6K*] Kan^r	(9)
RC12023	Δ*dgoR attλ*::[Kan promoterless-*venus oriR6K*] Kan^r	(9)
RC12018	BW25113 *attλ*::[Kan P$_{dgo}$-*venus oriR6K*] Kan^r	(9)
RC12020	Δ*dgoR attλ*::[Kan P$_{dgo}$-*venus oriR6K*] Kan^r	(9)
ECOR-24	Commensal *E. coli* strain	Typas lab (7)
RC16126	ECOR-24 *dgoR*::*kan* Kan^r	This work
ECOR-39	Commensal *E. coli* strain	Typas lab (7)
RC16105	ECOR-39 *dgoR*::*kan* Kan^r	This work
Plasmids		
pKD13	*oriR6K*, FRT-flanked Kan^r, pANTSγ PS1 PS4 Kan^r	(10)
pSIM5	pSC101 *ori* P$_L$-*gam-bet-exo* genes under the control of CI857 repressor (ts), Cam^r	Don Court (11)
pACYC177	p15A *ori* Amp^r Kan^r	New England Biolabs
pBS13	BW25113 *dgo* promoter and *dgoR*-6XHis in pACYC177, Amp^r Kan^r	(9)
pSW12	BW25113 *dgo* promoter and *dgoR*(L3F)-6XHis in pACYC177, Amp^r Kan^r	This work
pSW13	BW25113 *dgo* promoter and *dgoR*(K15R)-6XHis in pACYC177, Amp^r Kan^r	This work
pSW16	BW25113 *dgo* promoter and *dgoR*(H19Q)-6XHis in pACYC177, Amp^r Kan^r	This work
pSW10	BW25113 *dgo* promoter and *dgoR*(P24L)-6XHis in pACYC177, Amp^r Kan^r	This work
pSW28	BW25113 *dgo* promoter and *dgoR*(R71C)-6XHis in pACYC177, Amp^r Kan^r	This work
pSW29	BW25113 *dgo* promoter and *dgoR*(P92L)-6XHis in pACYC177, Amp^r Kan^r	This work
pSW11	BW25113 *dgo* promoter and *dgoR*(A128S)-6XHis in pACYC177, Amp^r Kan^r	This work
pSW26	BW25113 *dgo* promoter and *dgoR*(A152E)-6XHis in pACYC177, Amp^r Kan^r	This work
pSW8	BW25113 *dgo* promoter and *dgoR*(T180S)-6XHis in pACYC177, Amp^r Kan^r	This work
pMP18	BW25113 *dgo* promoter and *dgoR*(A186V)-6XHis in pACYC177, Amp^r Kan^r	This work
pMP19	BW25113 *dgo* promoter and *dgoR*(S220C)-6XHis in pACYC177, Amp^r Kan^r	This work
pSW15	BW25113 *dgo* promoter and *dgoR*(T222I)-6XHis in pACYC177, Amp^r Kan^r	This work
pSW35	ECOR-24 *dgo* promoter and *dgoR*-6XHis in pACYC177, Amp^r Kan^r	This work

pSW38	ECOR-24 *dgo* promoter and *dgoR*(C71R)-6XHis in pACYC177, Amp^r Kan^r	This work
pSW24	ECOR-39 *dgo* promoter and *dgoR*-6XHis in pACYC177, Amp^r Kan^r	This work
pSW25	ECOR-39 *dgo* promoter and *dgoR*(E152A)-6XHis in pACYC177, Amp^r Kan^r	This work
pRC10	pBR322 *ori*, −10 box of P_{trc} changed to P_{lac} in pTrc99a, Δ*Nco*I Amp^r	(12)
pBS2	BW25113 *dgoR*-6XHis in pRC10, Amp^r	(9)
pSW32	BW25113 *dgoR*(R71C)-6XHis in pRC10, Amp^r	This work
pSW30	BW25113 *dgoR*(A152E)-6XHis in pRC10, Amp^r	This work

‡CGSC, *E. coli* Genetic Stock Center

Table S3 *Primers used in this study*

Primers	Sequence (from 5' to 3')	Purpose	Reference
Primers used for cloning and verification			
BS23	ACCGGAATTCGAAGGAGATATACATATGACT CTCAATAAAACCGATCGCATTGTCATTAC	Forward primer for cloning BW25113 *dgoR*-6XHis in pRC10	(9)
BS24	CGTGGATCCTCA**GTGATGATGATGATGATG**T GTGATTTCCTTTAACCTTCGTGTCGAGC	Reverse primer for cloning BW25113 *dgoR*-6XHis in pRC10	(9)
BS25	GCTGTGGTATGGCTGTGCAGG	Sequencing/verification primer for cloning in pRC10	(9)
BS26	GCCAGGCAAATTCTGTTTTATCAG	Sequencing/verification primer for cloning in pRC10	(9)
BS97	ACCTGACGTCTATCTTTTGCCTGCGATAGC CCAG	Forward primer for cloning BW25113 *dgo* promoter and *dgoR*-6XHis in pACYC177	(9)
SW68	ACCTGACGTCTTTCAGATTTCTGCCCGACG CATG	Forward primer for cloning ECOR-24 or ECOR-39 *dgo* promoter and *dgoR*-6XHis in pACYC177	This work
BS24	CGTGGATCCTCA**GTGATGATGATGATGATG**T GTGATTTCCTTTAACCTTCGTGTCGAGC	Reverse primer for cloning BW25113, ECOR-24 or ECOR-39 *dgo* promoter and *dgoR*-6XHis in pACYC177	(9)
BS98	ATCAGTACCGACGGTGATATGG	Sequencing/verification primer for cloning in pACYC177	(9)
BS99	GGGTTATTGTCTCATGAGCGG	Sequencing/verification primer for cloning in pACYC177	(9)
Restriction sites are underlined.<br>Initiation codon of *dgoR* is highlighted in grey box.<br>6XHis tag sequence is shown in bold.			
Primers used for deleting genes			
SW61	CGATCGGGGTAAAGTAGAGAAGAACATACAGA GCACAAGGACTCTCCATGATTCCGGGGATCCG TCGACC	Forward primer for deleting ECOR-24 or ECOR-39 *dgoR*	This work
SW62	TCCCCAGTCAATTGCGATGTAGCGAGCTGTCA TGTGATTTCCTTTAACCTTGTAGGCTGGAGCTG CTTCG	Reverse primer for deleting ECOR-24 or ECOR-39 *dgoR*	This work
Primers used for verification of deletion strains			
SW66	CTGGTACTACAAAGTTGCCGCG	Forward primer for verification of ECOR-24 *dgoR*::*kan* or ECOR-39 *dgoR*::*kan*	This work
BS14	AGTGGTCGCCCTGATAAAGC	Reverse primer for verification of ECOR-24 *dgoR*::*kan* or ECOR-39 *dgoR*::*kan*	(9)
SAK1	GAGGCTATTCGGCTATGACTG	Forward primer specific to kanamycin cassette	(9)
SAK2	TTCCATCCGAGTACGTGCTC	Reverse primer specific to kanamycin cassette	(9)
Primers used for creating amino acid changes in BW25113 DgoR			
SW45/ SW46	GGACTCTCCATGACTTTTAATAAAACCGATCGC/ GCGATCGGTTTTATTAAAAGTCATGGAGAGTCC	Internal mutagenic primers for creating L3F mutation	This work
SW47/ SW48	GTCATTACGCTGGGTCGTCAGATCGTTCACGGC/ GCCGTGAACGATCTGACGACCCAGCGTAATGAC	Internal mutagenic primers for creating K15R mutation	This work

SW51/ SW52	GGTAAACAGATCGTTCAGGGCAAATACGTGCCA/ TGGCACGTATTTGCCCTGAACGATCTGTTTACC	Internal mutagenic primers for creating H19Q mutation	This work
SW29/ SW30	CACGGCAAATACGTGCTGGGCTCGCCGCTTCCG/ CGGAAGCGGCGAGCCCAGCACGTATTTGCCGTG	Internal mutagenic primers for creating P24L mutation	This work
SW81/ SW82	GCGTTTGTGGCACCGTGTAACCAGTGGAATTAC/ GTAATTCCACTGGTTACACGGTGCCACAAACGC	Internal mutagenic primers for creating R71C mutation	This work
SW79/ SW80	GAAAATGACTACGACCTACGGCTTATCAGTGCC/ GGCACTGATAAGCCGTAGGTCGTAGTCATTTTC	Internal mutagenic primers for creating P92L mutation	This work
SW35/ SW36	GCGCAGATTGAATCGAGCCTGAACGAGATGATT/ AATCATCTCGTTCAGGCTCGATTCAATCTGCGC	Internal mutagenic primers for creating A128S mutation	This work
SW71/ SW72	ATTCGCTACCACGAGGAGGTGCTGCAGTCGGTG/ CACCGACTGCAGCACCTCCTCGTGGTAGCGAAT	Internal mutagenic primers for creating A152E mutation	This work
SW31/ SW32	GCGGTTTTTGAACGAAGCTGGATGGGCGATGAG/ CTCATCGCCCATCCAGCTTCGTTCAAAAACCGC	Internal mutagenic primers for creating T180S mutation	This work
SW39/ SW40	TGGATGGGCGATGAGGTGAACATGCCGCAAACG/ CGTTTGCGGCATGTTCACCTCATCGCCCATCCA	Internal mutagenic primers for creating A186V mutation	This work
SW41/ SW42	CTTACCATGATCGCCTGCTCGACACGAAGGTTA/ TAACCTTCGTGTCGAGCAGGCGATCATGGTAAG	Internal mutagenic primers for creating S220C mutation	This work
SW37/ SW38	ATGATCGCCAGCTCGATTCGAAGGTTAAAGGAA/ TTCCTTTAACCTTCGAATCGAGCTGGCGATCAT	Internal mutagenic primers for creating T222I mutation	This work
Primers used for creating amino acid change in ECOR-24 DgoR			
SW85/ SW86	GCGTTTGTGGCACCGCGTAACCAGTGGAATTAC/ GTAATTCCACTGGTTACGCGGTGCCACAAACGC	Internal mutagenic primers for creating C71R mutation	This work
Primers used for creating amino acid change in ECOR-39 DgoR			
SW69/ SW70	ATTCGCTATCACGAGGCGGTGCTGCAGTCGGTG/ CACCGACTGCAGCACCGCCTCGTGATAGCGAAT	Internal mutagenic primers for creating E152A mutation	This work
Mutated codon is underlined.			
Oligonucleotides used for making Cy5-labeled dsDNA for EMSA			
GA149	Cy5'-GTGATCTAAATTGTAGTACAACAATATAA GTTTGTACTACATTACACGCACG	5'-end Cy5-labeled single-stranded oligonucleotide	(1)
GA150	CGTGCGTGTAATGTAGTACAAACTTATATTGTT GTACTACAATTTAGATCAC	Unlabeled single-stranded oligonucleotide	(1)

SUPPLEMENTAL DATASET

Dataset S1. *Prevalence data for P24L, R71C, P92L, and A152E variations.* (A) Prevalence of the four variations across *E. coli* and Enterobacterales Identical Protein Group datasets as depicted in Columns C and D. Numbers within the bracket represent the total number of identical proteins identified amongst the total Identical Protein Groups shown outside the bracket. Columns E, F, and G also provide natural isolate information from Table S1 (7). (B-E) These four sheets show the information of all identified strains with respective variations, i.e., P24L (B), R71C (C), P92L (D), and A152E (E). Column A shows the Identical Protein Group dataset ID, which will be the same for all identical proteins. Other columns, B to K, show information for each strain. Column L shows the presence within *E. coli*, Enterobacterales, or both datasets.

The Supplemental Dataset S1 is provided as a separate .xlsx file.

SUPPLEMENTAL REFERENCES

1. Arya G, Pal M, Sharma M, Singh B, Singh S, Agrawal V, Chaba R. 2021. Molecular insights into effector binding by DgoR, a GntR/FadR family transcriptional repressor of D-galactonate metabolism in *Escherichia coli*. 4. Mol Microbiol 115:591–609.

2. Singh S, Arya G, Mishra R, Singla S, Pratap A, Upadhayay K, Sharma M, Chaba R. 2025. Molecular mechanisms underlying allosteric behavior of *Escherichia coli* DgoR, a GntR/FadR family transcriptional regulator. Nucleic Acids Res 53:gkae1299.

3. Humphrey W, Dalke A, Schulten K. 1996. VMD: visual molecular dynamics. 1. J Mol Graph 14:33–38, 27–28.

4. Grant BJ, Rodrigues APC, ElSawy KM, McCammon JA, Caves LSD. 2006. Bio3d: an R package for the comparative analysis of protein structures. 21. Bioinformatics 22:2695–2696.

5. Eargle J, Luthey-Schulten Z. 2012. NetworkView: 3D display and analysis of protein·RNA interaction networks. 22. Bioinformatics 28:3000–3001.

6. Girvan M, Newman MEJ. 2002. Community structure in social and biological networks. 12. Proc Natl Acad Sci USA 99:7821–7826.

7. Galardini M, Koumoutsi A, Herrera-Dominguez L, Cordero Varela JA, Telzerow A, Wagih O, Wartel M, Clermont O, Denamur E, Typas A, Beltrao P. 2017. Pheno type inference in an *Escherichia coli* strain panel. eLife 6:e31035.

8. Baba T, Ara T, Hasegawa M, Takai Y, Okumura Y, Baba M, Datsenko KA, Tomita M, Wanner BL, Mori H. 2006. Construction of *Escherichia coli* K-12 in-frame, single-gene knockout mutants: the Keio collection. 1. Mol Syst Biol 2:2006.0008.

9. Singh B, Arya G, Kundu N, Sangwan A, Nongthombam S, Chaba R. 2019. Molecular and functional insights into the regulation of D-galactonate metabolism by the transcriptional regulator DgoR in *Escherichia coli*. 4. J Bacteriol 201:e00281-00218.

10. Datsenko KA, Wanner BL. 2000. One-step inactivation of chromosomal genes in *Escherichia coli* K-12 using PCR products. 12. Proc Natl Acad Sci USA 97:6640–6645.

11. Datta S, Costantino N, Court DL. 2006. A set of recombineering plasmids for gra m-negative bacteria. Gene 379:109–115.

12. Chaba R, Grigorova IL, Flynn JM, Baker TA, Gross CA. 2007. Design principles

of the proteolytic cascade governing the sigmaE-mediated envelope stress response in *Escherichia coli*: keys to graded, buffered, and rapid signal transduction. 1. Genes Dev 21:124–136.
